# The Loss of Lam2 and Npr2-Npr3 Diminishes the Vacuolar Localization of Gtr1-Gtr2 and Disinhibits TORC1 Activity in Fission Yeast

**DOI:** 10.1371/journal.pone.0156239

**Published:** 2016-05-26

**Authors:** Ning Ma, Yan Ma, Akio Nakashima, Ushio Kikkawa, Tomoyuki Furuyashiki

**Affiliations:** 1 Division of Pharmacology, Graduate School of Medicine, Kobe University, Kobe, Japan; 2 Biosignal Research Center, Kobe University, Kobe, Japan; Kinki University School of Pharmaceutical Sciences, JAPAN

## Abstract

In mammalian cells, mTORC1 activity is regulated by Rag GTPases. It is thought that the Ragulator complex and the GATOR (GAP activity towards Rags) complex regulate RagA/B as its GDP/GTP exchange factor (GEF) and GTPase-activating protein (GAP), respectively. However, the functions of components in these complexes remain elusive. Using fission yeast as a model organism, here we found that the loss of Lam2 (SPBC1778.05c), a homolog of a Ragulator component LAMTOR2, as well as the loss of Gtr1 or Gtr2 phenocopies the loss of Npr2 or Npr3, homologs of GATOR components Nprl2 or Nprl3, respectively. These phenotypes were rescued by TORC1 inhibition using pharmacological or genetic means, and the loss of Lam2, Gtr1, Gtr2, Npr2 or Npr3 disinhibited TORC1 activity under nitrogen depletion, as measured by Rps6 phosphorylation. Consistently, overexpression of GDP-locked Gtr1^S20L^ or GTP-locked Gtr2^Q60L^, which suppress TORC1 activity in budding yeast, rescued the growth defect of Δ*gtr1* cells or Δ*gtr2* cells, respectively, and the loss of Lam2, Npr2 or Npr3 similarly diminished the vacuolar localization and the protein levels of Gtr1 and Gtr2. Furthermore, Lam2 physically interacted with Npr2 and Gtr1. These findings suggest that Lam2 and Npr2-Npr3 function together as a tether for GDP-bound Gtr1 to the vacuolar membrane, thereby suppressing TORC1 activity for multiple cellular functions.

## Introduction

Target of rapamycin (TOR) is a serine/threonine kinase, and plays fundamental roles in regulating cell growth and metabolism by coordinating diverse cellular processes including transcription, translation and autophagy [1, 2]. Mammalian cells express a single TOR isoform mTOR, which forms two types of protein complexes named mTORC1 and mTORC2 [1, 2]. In fission yeast, there are two TOR isoforms Tor2 and Tor1, each of which is included in TORC1 or TORC2 [3, 4]. TORC1 is activated by the GTP-bound form of Rheb small GTPase. In mammalian cells, growth factors, energy status and oxygen levels increase the GTP-bound form of Rheb by inhibiting TSC2, a GTPase activing protein (GAP) for Rheb, and consequently activate mTORC1. However, TSC2 ortholog is expressed in fission yeast, but not in budding yeast. Studies in mammalian cells have revealed that amino acids activate mTORC1 through the Rag GTPase superfamily (RagA, B, C and D) in a TSC2-independent manner [5]. RagA or RagB forms a complex with RagC or RagD, and mTORC1 is activated when RagA or RagB is bound to GTP and RagC or RagD is bound to GDP. Rag GTPases regulate the localization of mTORC1 to lysosomes, which may promote the association of mTORC1 with the GTP-bound form of Rheb [6, 7]. The Rag GTPases are conserved across species, and yeast cells express these orthologs named Gtr1 and Gtr2 (S1 Fig).

Rag GTPases are thought to be regulated by GDP/GTP exchange factors (GEFs) and GAPs similarly to other small GTPases. GAPs for RagA and RagB are conserved from yeast to mammalian cells (S1 Fig). In budding yeast, the octameric Seh1-associated complex (SEAC) was identified as a negative regulator for TORC1 [8, 9]. SEAC is composed of two subcomplexes SEACIT (Npr2-Npr3-Iml1) and SEACAT (Seh1-Sea2-Sea3-Sea4-Sec13), and Iml1 in SEACIT acts as a GAP for Gtr1 [10, 11]. Mammalian cells express the GATOR (GAP activity towards Rags) complex equivalent to SEAC. GATOR is composed of two subcomplexes GATOR1 (Nprl2-Nprl3-DEPDC5) and GATOR2 (WDR59-WDR24-Mios-Seh1L-Sec13), each of which corresponds to SEACIT and SEACAT in yeast cells, respectively. GATOR1, especially DEPDC5, has a GAP activity toward RagA and RagB, and inhibits mTORC1 under low amino acid condition. When the level of amino acids becomes high, GATOR2 is thought to inhibit the GAP activity of GATOR1, thus activating mTORC1 signaling [12, 13]. However, whether Npr2-Npr3 or Nprl2-Nprl3 functions only as components of a GAP for Gtr1 or RagA has not been proven.

Molecular identity of a GEF for Rag GTPases remains much less understood (S1 Fig). Studies in budding yeast identified the EGO (Exit from rapamycin-induced growth arrest) complex as a positive regulator for TORC1. The EGO complex is composed of Ego1 and Ego3 together with Gtr1 and Gtr2. Loss of Ego1 or Ego3 impairs recovery from rapamycin-induced growth arrest, and reduces phosphorylation of Sch9 as a readout of TORC1 activity [14–16]. In mammalian cells, pentameric Ragulator complex composed of LAMTOR1, 2, 3, 4 and 5 was identified as a tether of Rag GTPases and mTORC1 to lysosomal membranes [17]. This complex, but not individual components, has been shown to function as a GEF for RagA and RagB. Whereas LAMTOR1 and LAMTOR2-LAMTOR3 are thought to function similarly to Ego1 and Ego3, respectively [16], studies in budding and fission yeast have shown that Vam6, a vacuolar ATPase, rather than the EGO complex functions as a GEF for Gtr1 [14, 18]. Therefore, in yeast cells, whether each component of Ragulator functions to promote a GEF activity for Rag GTPases or may function as a tether for Rag GTPases also remains elusive.

Using fission yeast, we performed a genome-wide screen to identify negative regulators of TORC1 by isolating mutants that phenocopy Δ*tsc2*, and found Npr2 as a TSC2-independent negative regulator of TORC1 [19]. In the same screen, we also identified Lam2 (SPBC1778.05c), a homolog of a Ragulator component LAMTOR2, as another candidate. Here we found that the loss of Lam2 or Gtr1-Gtr2 increases TORC1 activity and consequently phenocopies the loss of Npr2-Npr3 in multiple cellular functions. Our findings further suggest that Lam2 and Npr2-Npr3 form a physical complex with Gtr1 and functions as a tether of GDP-bound Gtr1 to the vacuolar membrane, thereby suppressing TORC1 activity.

## Materials and Methods

### Yeast Strains, Growth Media, Drugs and General Methods

The *Schizosaccharomyces pombe* strains used in this study are listed in S1 Table. The complete medium YPD (yeast-extract–peptone–dextrose), YES and the minimal medium EMM were described previously [20, 21]. Through this study, we found that some of the previously reported phenotypes of Δ*gtr1* cells, Δ*gtr2* cells and Δ*npr2* cells might be due to the secondary mutation irrelevant to the loss of the respective genes (see the Results section). Since we suspect that these secondary mutations might be introduced to overcome the impeded cell growth of these cells on YES or YPD medium, we decided to maintain Δ*lam2* cells, Δ*gtr1* cells, Δ*gtr2* cells, Δ*npr2* cells and Δ*npr3* cells in EMM medium in order to avoid such secondary mutations, just prior to the experiments. Gene disruptions are indicated by the gene symbol preceded by Δ (for example, Δ*gtr1*). Proteins are denoted by Roman letters with only the first letter capitalized (for example, Gtr1). Drugs were obtained from the following sources: rapamycin (Toronto Research Chemicals), Torin-1 (Tocris), canavanine (Tokyo Chemical Industry), anhydrotetracycline (ahTet) (Funakoshi). Database searches were performed using Pombe community database PomBase (http://www.pombase.org).

### Plasmids

The plasmids to express the *lam2*^+^, *gtr2*^+^ and *gtr1*^+^genes under the respective native promoters were generated using conventional methods. Briefly, the *lam2*^*+*^, *gtr2*^*+*^ or *gtr1*^*+*^ gene was amplified by polymerase chain reaction (PCR) with genomic DNA of wild-type cells as a template. For *lam2*^*+*^, the sense primer was (4511) 5’-CGG GAT CCA TTT GTT TCG ACT TAA CTA TAG-3’ and the antisense primer was (4001) 5’-CGG GAT CCG CGG CCG CTT AGA CTG GTT TTC CAA GCG TGG-3’. For *gtr2*^*+*^, the sense primer was (4004) 5’-CGG GAT CCA TGA AGC CTA GAA AGA TTA TTT TAA TG-3’ and the antisense primer was (4005) 5’-CGG GAT CCG CGG CCG CCT GTT CTA GGT GAG AAA ATG GAC-3’. For *gtr1*^*+*^, the sense primer was (4907) 5’-CGG GAT CCG TGG AAA GCT CAT CCA AGA TG-3’ and the antisense primer was (4908) 5’-CGG GAT CCG TAC CGA TTA TGC TAT GTT TG-3’. The amplified product containing *lam2*^+^ or *gtr2*^+^ was digested with BamHI and NotI, and the product containing *gtr1*^+^ was digested with BamHI. The resulting fragment was subcloned into the BamHI/NotI or BamHI sites of a multicopy expression vector pKB1037, which was generated by inserting an autonomously replicating sequence (*ARS*) and LEU2 marker into the BglII site of pKB1030 [22]. The resulted plasmids expressing the *lam2*^+^, *gtr2*^+^ and *gtr1*^+^ genes are registered as pKB8727, pKB8746 and pKB9042, respectively.

The plasmids to express the *npr2*^+^ and *lam2*^+^ genes fused with GST or GFP(S65T) under the thiamine-repressible *nmt1* promoter [23] were generated as follows. The complete open reading frame (ORF) of the *npr2*^*+*^ gene was amplified using a sense primer (3882) 5’-CGG GAT CCA TGG AGT ATT CTG AAG AGG G-3’ and an antisense primer (3883) 5’-CGG GAT CCG CGG CCG CTC ATA CAT AAA TAA AAC AGG C-3’. The ORF of the *lam2*^*+*^ gene was amplified using a sense primer (4000) 5’-CGG GAT CCA TGA TTA AGC CAA AGA AGT TG-3’ and an antisense primer (4001) 5’-CGG GAT CCG CGG CCG CTT AGA CTG GTT TTC CAA GCG TGG-3’. The amplified products containing the *npr2*^+^ or *lam2*^+^ gene were digested with BamHI and NotI, and were ligated to the BamHI/NotI sites at the N-terminus of GST in pDS473aL [24] or at the N-terminus of GFP in a plasmid which was generated by replacing the HA tag of pSLF173L [24] with the GFP(S65T) ORF. The resultant plasmid pREP1-GST-Npr2 and pREP1-GFP-Npr2 were registered as pKB8567 and pKB8568, respectively. The resultant plasmid pREP1-GST-Lam2 and pREP1-GFP-Lam2 were registered as pKB8795 and pKB8682, respectively. The chromosome-borne GFP-Lam2 or GFP-Npr2 strains were generated with conventional methods [25]. The fragment containing the *nmt1* promoter and the ORF of GFP-Lam2 or GFP-Npr2 was removed from the plasmids generated above, and were subcloned into an integration vector pKB3282, which was generated by inserting *ura4*^+^ marker into the BglII site of pKB1030 [22], and was integrated into the chromosome at the non-functional *ura4* gene locus of KP1248.

The plasmids expressing Gtr1, Gtr1^Q61L^ and Gtr1^S20L^ fused with GST and Gtr1 fused with GFP(S65T) were generated as follows. The ORF of *gtr1*^*+*^ gene was amplified using a sense primer (4002) 5’-CGG GAT CCA TGA AGC CTA GAA AGA TTA TTT TAA TG-3’ and an antisense primer (4003) 5’-CGG GAT CCG CGG CCG CCT GTT CTA GGT GAG AAA ATG GAC-3’. The amplified products containing the *gtr1*^+^ gene were digested with BamHI/NotI, and were ligated to the BamHI/NotI sites of pBlueScript SK(+) (Stratagene) and regesitered as pKB8676. Using the Quick Change mutagenesis kit (Stratagene), the ORF of *gtr1*^Q61L^ (pKB8910) was generated with pKB8676 as a template, a sense primer (4755) 5’-CTA TGG GAT TGC GGT GGA CTC GAG GCG TTC ATG GAA AAC-3’ and an antisense primer (4756) 5’-GAT ACC CTA ACG CCA CCT GAG CTC CGC AAG TAC CTT TTG-3’. The ORF of *gtr1*^S20L^ (pKB8911) was generated with pKB8676 as a template, a sense primer (4745) 5’-GCA AAA GTT CAA TGA GAC TCA TAG TTT TTA GCA ATT ATG -3’ and an antisense primer (4746) 5’-CGT TTT CAA GTT ACT CTG AGT ATC AAA AAT CGT TAA TAC-3’. To generate pREP1-Gtr1-GST, pREP1-Gtr1^Q61L^-GST and pREP1-Gtr1^S20L^-GST, the ORF of *gtr1*^+^, *gtr1*^Q61L^ or *gtr1*^S20L^ was removed from the pKB8676, pKB8910 or pKB8911, respectively, with BamHI and NotI, and ligated into the BglII/NotI sites at the C-terminus of GST in pDS472aL [24] (registered pKB2437 in our lab). To generate pREP1-Gtr1-GFP (pKB8702), pKB8676 was digested with BamI/NotI, and the resultant fragment containing the ORF of *gtr1*^+^ was ligated into the BglII/NotI sites of the C-terminus of GFP(S65T) in pKB2728, which was generated by replacing the HA tag of pSLF172L [24] with the GFP(S65T) ORF.

The plasmids expressing Gtr2, Gtr2^Q60L^ and Gtr2^S17L^ fused with GST and Gtr2 fused with GFP(S65T) were generated as follows. The ORF of *gtr2*^*+*^ gene was amplified using a sense primer (4004) 5’-CGG GAT CCA TGA AGC CTA GAA AGA TTA TTT TAA TG-3’ and an antisense primer (4005) 5’-CGG GAT CCG CGG CCG CCT GTT CTA GGT GAG AAA ATG GAC-3’. The amplified products containing the *gtr2*^+^ gene were digested with BamHI, and were ligated to the BamHI site of pGEM7Zf (Promega) and regesitered as pKB8634. Using the Quick Change mutagenesis kit (Stratagene), the ORF of *gtr2*^Q60L^ (pKB8905) was generated with pKB8634 as a template, a sense primer (4749) 5’-GTT TGG GAT TTC CCT GGC CTC GTA GAT GTG TTT GAT GC-3’, and an antisense primer (4750) 5’-CAA ACC CTA AAG GGA CCG GAG CAT CTA CAC AAA CTA CG-3’. The ORF of *gtr2*^S17L^ (pKB8904) was generated with pKB8676 as a template, a sense primer (4747) 5’-GAC TTC GCC GTA GTG GCA AGC TCT CAA TCC AAA AGG TGG-3’, and an antisense primer (4748) 5’-CTG AAG CGG CAT CAC CGT TCG AGA GTT AGG TTT TCC ACC-3’. To generate pREP1-Gtr2-GST, pREP1-Gtr2^Q60L^-GST and pREP1-Gtr2^S17L^-GST, the ORF of *gtr2*^+^, *gtr2*^Q60L^ and *gtr2*^S17L^ were removed from pKB8634, pKB8905, and pKB8904, respectively, with BglII/NotI, and ligated into the BglII/NotI sites at the C-terminus of GST of pKB2437. To generate pREP1-Gtr2-GFP (pKB9133), pKB8634 was digested with BglII/NotI, and the ORF of *gtr2*^+^ was ligated into the BglII/NotI sites of the C-terminus of GFP in pKB2728.

### Gene Deletion

Disruptions of *gtr1*^+^ and *gtr2*^+^ with the *kanMX* and *hphMX* cassettes, respectively, were performed by the PCR-base direct chromosomal integration methods [26, 27]. For *gtr1*^+^, the sense primer was 5’-CAC GAA TTC ACT CAC AAT TGC AGT GAC AGC TGT TTT GTA GAA TTT TAT AAA CTA ATT GCT TTA CCC TTA ATC TCA AGA TAC GGA TCC CCG GGT TAA TTA A-3’, and the antisense primer was 5’-CTA GGT AAT TAC AGC AAC AAG AGT AAA ATA CAT TAG ATC CAC ATT TTT TAC GAT TAT CAA AGA AAA AAG AAC AAG ATT GAG AAT TCG AGC TCG TTT AAA C-3’. For *gtr2*^*+*^, the sense primer was 5’-ATG GGG TGT CTA GTC ACC ACC AGA GAA ACG CAC AAT TTA CCA CGT AGA AAC TTA CTT TTG TTA ATG ACT TTG ATG AAA TAC GGA TCC CCG GGT TAA TTA A-3’, and the antisense primer was 5’-AAA TCA ACA CAG AAG CGA TTG ATT TCA AAG GTT TCA TGT TCT AGG TGA GAA AAT GGA CTG AAT AGC TGT CTG TAG ACA TTG AAT TCG AGC TCG TTT AAA C-3’. The disruption of the resultant strains was confirmed by genomic PCR, and the strains were subjected to back-crossing.

### Inducible Expression of the *lam2*^*+*^ and *gtr2*^*+*^ Genes

A two-step tetracycline-regulated system [28] was applied to control the expression of Lam2 or Gtr2. Briefly, as the first step, the promoter of *lam2*^+^ or *gtr2*^+^ was replaced by a tetracycline-regulated promoter (*tet*O_7_-TATA_*CYC1*_). For this purpose, the *hphMX6*-*tet*O_7_-TATA_*CYC1*_-FLAG_3_ cassette flanked with a distal region of the *lam2*^+^ or *gtr2*^+^ promoter (approximately 80 bp) and the initial portion of the open reading frame of *lam2*^+^ or *gtr2*^+^ (approximately 80 bp) was generated by PCR using the plasmid pFA6a-*hphMX6-tet*O_7_-TATA_*CYC1*_-FLAG_3_ (Addgene, #41020) as a template. For *lam2*^+^, the sense primer was (5004) 5’-AAC AGG TTA AAT CGT TAT TGC CGA TTG GAC TTT AAA ATA CAT ATT GCT ACC TTT ACT GCT ATC TAA GTT TAA ATT CGA AGG AAT TCG AGC TCG TTT AAA C-3’ and the antisense primer was (5005) 5’-CAT GAT AGA CGG TAC AGT TTC CTC AAC TGC CTG TTT CAT CAA CGA CGA CAA CTT CTT TGG CTT AAT CAT TCC GCC TCC TTT ATC ATC ATC GTC CTT ATA G-3’. For *gtr2*^+^, the sense primer was (5002) 5’-ATG GGG TGT CTA GTC ACC ACC AGA GAA ACG CAC AAT TTA CCA CGT AGA AAC TTA CTT TTG TTA ATG ACT TTG ATG AAA TAG AAT TCG AGC TCG TTT AAA C-3’ and the antisense primer was (5003) 5’-CAC CAC CTT TTG GAT TGA TGA CTT GCC ACT ACG GCG AAG TCC CAT TAA AAT AAT CTT TCT AGG CTT CAT TCC GCC TCC TTT ATC ATC ATC GTC CTT ATA G-3’. The amplified product was transformed into yeast strain KP456. Stable integrants were selected on YES plates containing 100 μg/ml hygromycin, confirmed by genomic PCR and registered as KP6643 (*tet*O_7_-TATA_*CYC1*_-FLAG_3_-*lam2*:*hphMX6*), and KP6636 (*tet*O_7_-TATA_*CYC1*_-FLAG_3_-*gtr2*:*hphMX6*), respectively. As the second step, a plasmid expressing TetR-based transcription repressor (pDM291*-tet*R*-tup11*Δ*70*, Addgene, #41027) was digested with BamHI and transformed into KP6643 and KP6636 generated above. Stable integrants were selected on EMM medium lacking uracil. In these integrants, the expression of Lam2 or Gtr2 was suppressed without tetracycline analogue ahTet, and was induced by the presence of ahTet (2.5 μg/ml).

### Co-precipitation and Immunoblot Analyses

Co-precipitation and immunoblot analyses were performed as previously described [29–31]. For co-precipitation, the cells were grown to mid-log phase without thiamine to induce expression of proteins to be analyzed under the thiamine-repressible *nmt1* promoter. Then the resulted cells were collected and resuspended in 450 μl of ice-cold lysis buffer (50 mM Tris-HCl, pH 8, containing 2 mM EDTA, 1 mM dithiothreitol, 150 mM NaCl, 1% Triton X-100, 1 mM phenylmethylsulfonyl fluoride) containing Halt Protease Inhibitor Cocktail, EDTA-Free (Thermo scientific). The cells were disrupted with microbeads (0.2g). Then the microbeads and cellular debris were removed by centrifugation at 15,000 rpm for 15 min at 4°C. To precipitate GST-tagged proteins, the protein extracts were rotated with glutathione sepharose 4B beads at 4°C for 2 hours. Glutathione sepharose 4B beads were washed with ice-cold wash buffer (50 mM Tris-HCl, pH 8, containing 2 mM EDTA, 1 mM dithiothreitol, 500 mM NaCl, 1% Triton X-100,), eluted with 1x SDS-PAGE sample buffer with 2-mercaptoethanol (Nacalai), and subjected to immunoblot analysis. For detection of Rps6 phosphorylation, Gtr1-GFP and Gtr2-GFP, cell lysates were prepared using a 1.85M NaOH containing β-mercaptoethanol (7.5% v/v), and was neutralized with 50% trichloroacetic acid (TCA). The resultant lysates were mixed with 2x SDS-PAGE sample buffer.

The resultant samples were subjected to SDS-PAGE with 10% precast polyacrylamide gels (Nacalai). Proteins were then transferred onto PVDF membrane. After blocking, the membrane was incubated with rabbit anti-GFP antibody ([25], anti-GST antibody [25], mouse anti-α-tubulin antibody (clone B5-1-2, Sigma-Aldrich) or rabbit Phospho-(Ser/Thr) Akt substrate antibody (#9611, Cell Signaling Technology), which recognizes the (R/K)X(R/K)XX(pT/pS) motif and is known to cross-react Rps6 phosphorylation [30]. The resultant membrane was further incubated with HRP-conjugated anti-rabbit or anti-mouse antibody (Cell Signaling Technology). Signals were detected using Clarity Western ECL Substrate (BioRad) and autoradiography films (Fujifilm) or Luminoimage Analyzer (LAS1000, Fujifilm).

### Quantitative RT-PCR

Quantitative RT-PCR was performed as previously described [21]. The cells were cultuted to mid-log phase and subjected to RNeasy Mini kit (Qiagen) followed by on-column deoxyribonuclease digestion (Qigen). The resultant RNA was used to synthesize cDNA using the High Capacity cDNA Reverse Transcription Kit (ABI). The resultant cDNA was subjected to quantitative PCR with the SYBR Green PCR Master Mix (ABI). The fluorescent signals were detected and analyzed with the Applied Biosystems 7500 Real-Time PCR System (ABI). The mRNA levels of *isp5*^+^ and *cat1*^+^ were normalized to those of *act1*^+^ according to the comparative CT method, and were statistically analyzed. The *isp5*^+^ primers were 5’-TCG GTG TAC GAG GTT ATG GT-3’ and 5’-GGT GGA AAA GAC AGA GCA GA-3’. The *cat1*^+^ primers were 5’-GTT TCG ACA TGG GTT CAA AG-3’ and 5’-AAC TTG CTT AAC GGC ATG AG-3’. The *act1*^+^ primers were 5’-ATC CAA CCG TGA GAA GAT GA-3’ and 5’-ACC ATC ACC AGA GTC CAA GA-3’.

### Fluorescent Imaging

The yeast strains expressing Gaf1-YFP or Cat1-GFP under its native promoter and those expressing Gtr1-GFP or Gtr2-GFP under *nmt1* promoter were generated and observed as described previously [32]. Briefly, the cells were cultured to mid-log phase, and mounted on glass slides. Fluorescent images from live cells were acquired using a microscope (Axioskop 2 Plus; Carl Zeiss) equipped with an alpha Plan-Fluor 100x/N.A.1.45 oil objective lens (Carl Zeiss) and a Visualix VTCH1.4ICE digital camera in combination with the Iscapture software version 4.0.1 (Visualix). Differential interference contrast (DIC) images were also acquired. Fluorescent images were processed using Adobe Photoshop CS6 only for illustrative purposes.

### Luciferase Reporter Assay

A Renilla luciferase reporter plasmid with the *isp5*^+^ promoter (pKB8527) was constructed, and the luciferase reporter assay was performed, as described previously [19]. Briefly, the cells were transformed with this plasmid and cultured to mid-log phase. The resultant cells were added with coelenterazine (Promega), and the bioluminescence was real-time measured as relative light units (RLU) at 27°C every minute with a microplate luminometer (AB-2350, ATTO). The peak RLU value during the 2-hour observation period was obtained and statistically analyzed.

### Statistical Analyses

Data are shown as means ± SEM. Unpaired t-test was used to statistically analyze the difference between two groups. One-way ANOVA followed by Tukey’s multiple compoarison tests was performed to statistically analyze the differences among more than two groups. The *P* values less than 0.05 are considered to be significant. Statistical analyses were performed with Prism 6 (GraphPad).

## Results

### Δ*lam2* Cells, Δ*gtr1* Cells and Δ*gtr2* Cells Show Growth Defect in a TORC1-dependent Manner, Similarly to Δ*npr2* Cells and Δ*npr3* Cells

**Fig 1:**
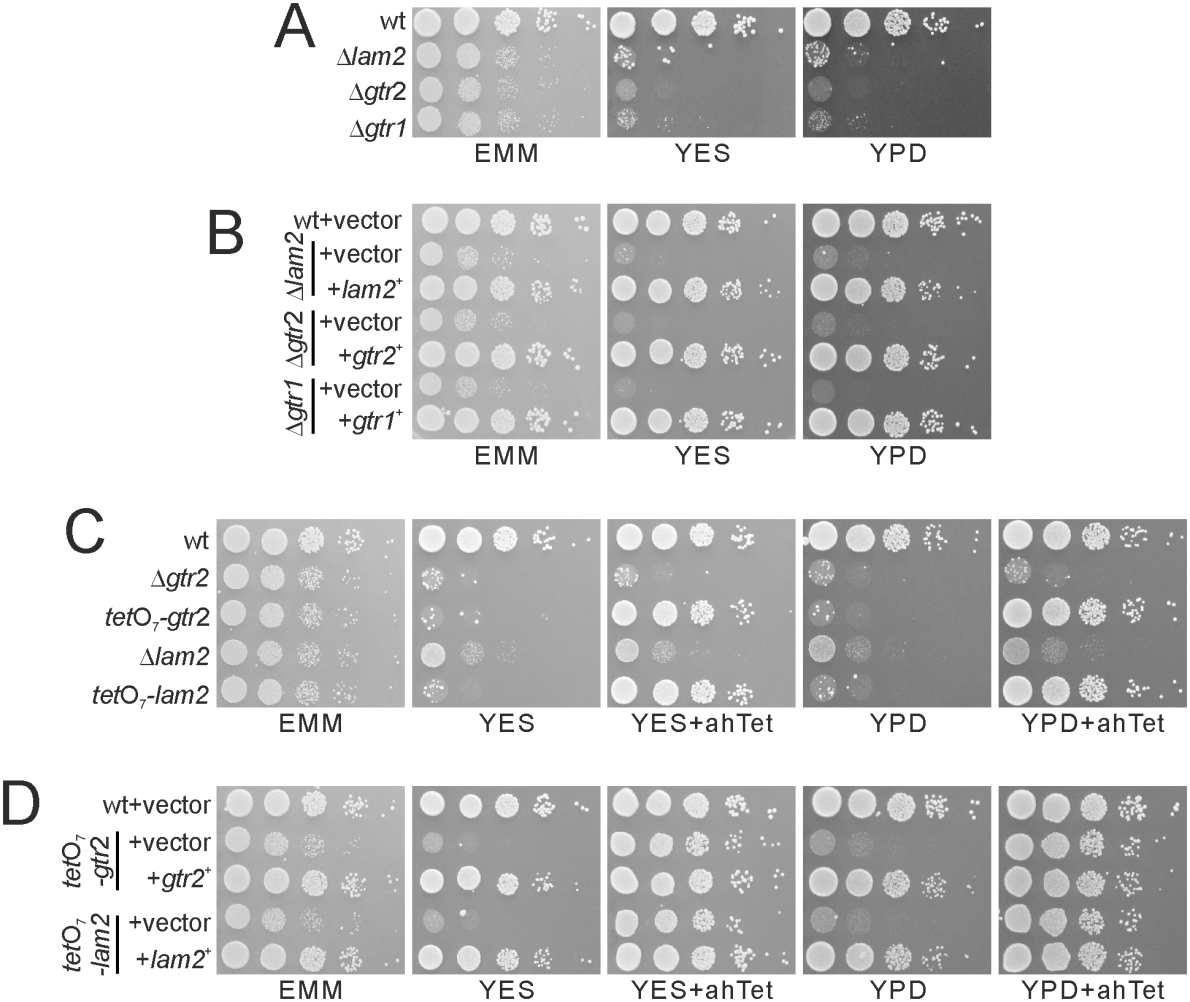
The deficits in Lam2, Gtr1 and Gtr2 cause growth defects. (A) Δ*lam2* cells, Δ*gtr1* cells and Δ*gtr2* cells showed growth defects. Prototrophic wild-type cells (wt, KP5080), Δ*lam2* cells (KP6578), Δ*gtr1* cells (KP6573) and Δ*gtr2* cells (KP6571) were cultured to mid-log phase, and adjusted to 0.3 in OD660. The cells were serially diluted by 10 fold and spotted onto the indicated plates, and were then incubated at 27°C on EMM plates for 4 days and on YES or YPD plates for 3 days. (B) Growth defects of Δ*lam2* cells, Δ*gtr2* cells and Δ*gtr1* cells were rescued by overexpression of the respective genes. The indicated leucine auxotrophic Δ*lam2* cells (KP6607), Δ*gtr2* cells (KP6608) and Δ*gtr1* cells (KP6688) transformed with the control vector (pKB1037) or the plasmid containing the *lam2*^+^ (pKB8727), *gtr2*^+^ (pKB8746) or *gtr1*^+^ (pKB9042) gene were dropped and incubated as described in Fig 1A. (C) *tet*O_7_-*lam2* cells and *tet*O_7_-*gtr2* cells phenocopied Δ*lam2* cells and Δ*gtr2* cells. The Δ*gtr2* cells (KP6571), *tet*O_7_-*gtr2* cells (KP6645), Δ*lam2* cells (KP6578) and *tet*O_7_-*lam2* cells (KP6650) were spotted onto the indicated plates with or without 2.5 μg/ml ahTet, and then incubated as described in Fig 1A. (D) Growth defects of *tet*O_7_-*lam2* cells and *tet*O_7_-*gtr2* cells were rescued by overexpression of the respective genes. The *tet*O_7_-*gtr2* cells (KP6641) transformed with the control vector (pKB1037) or the plasmid containing the *gtr2*^+^ gene (pKB8746), or the *tet*O_7_-*lam2* cells (KP6648) transformed with the control vector (pKB1037) or the plasmid containing the *lam2*^+^ gene (pKB8727) were spotted and incubated as described in Fig 1A.

Using knockout cell library purchased from Bioneer Corporation [33], we performed a genome-wide screen to identify negative regulators of TORC1 by isolating mutants that phenocopy Δ*tsc2* in terms of canavanine resistance. We found that the loss of *lam2*^+^/SPBC1778.05c encoding an ortholog of human LAMTOR2, a component of Ragulator that promotes mTORC1 activity [13], shows canavanine resistance (data not shown). Since Δ*lam2* cells (registered as KP92662) in this library is auxotrophic for leucine, uracil and adenine, we first obtained prototrophic Δ*lam2* cells by random spore analysis on EMM plates. The resultant Δ*lam2* cells grew slowly on EMM plates, and failed to grow on both YES and YPD plates (Fig 1A). To perform rescue experiments, we further obtained Δ*lam2* cells auxotrophic for leucine. Overexpression of the *lam2*^*+*^ gene completely rescued the growth defect of Δ*lam2* cells (Fig 1B), confirming that the loss of Lam2 causes this growth defect.

To confirm the phenotypes due to the loss of Lam2, we replaced the native promoter of *lam2*^+^ by *tet*O_7_, a tetracycline-inducible promoter [28], and temporally regulated expression of Lam2. In the resultant cells (*tet*O_7_-*lam2*), the expression of Lam2 was inhibited without ahTet, a tetracycline analog. Similarly to Δ*lam2* cells, *tet*O_7_-*lam2* cells grew slowly on EMM plates and failed to grow on YES or YPD plates without ahTet (Fig 1C, *tet*O_7_-*lam2*). In the presence of ahTet, the expression of Lam2 was induced, and *tet*O_7_-*lam2* cells came to grow similarly to wild-type cells (Fig 1C, *tet*O_7_-*lam2*). Overexpression of the *lam2*^*+*^ gene completely rescued the gowth defect of *tet*O_7_-*lam2* cells in the absence of ahTet (Fig 1D, *tet*O_7_-*lam2*). These results indicate that the growth defect on YES and YPD media is a *bona fide* phenotype of *lam2* deletion.

Whereas LAMTOR2 is a component of Ragulator that promotes the GDP/GTP exchange of Rag GTPases, it was reported that Δ*gtr1* cells and Δ*gtr2* cells normally grow on YES plates [19, 34]. To re-examine this phenotype, we generated new strains of Δ*gtr1* cells and Δ*gtr2* cells, and found that these mutant cells grew slowly on EMM plates, and failed to grow on both YES and YPD plates, similarly to Δ*lam2* cells (Fig 1A). Overexpression of the *gtr2*^*+*^ or *gtr1*^*+*^ gene rescued the growth defect of Δ*gtr2* or Δ*gtr1* cells, respectively (Fig 1B), confirming that *gtr2* or *gtr1* deletion is the cause for the growth defect. To further confirm the phenotype due to the loss of Gtr2, we generated *tet*O_7_-*gtr2* cells, in which the native *gtr2*^+^ promoter was replaced by *tet*O_7_ promoter. Similarly to Δ*gtr2* cells, *tet*O_7_-*gtr2* cells grew slowly on EMM plates and failed to grow on YES and YPD plates without ahTet (Fig 1C, *tet*O_7_-*gtr2*). In the presence of ahTet, the expression of Gtr2 was induced, and *tet*O_7_-*gtr2* cells came to grow similarly to wild-type cells (Fig 1C, *tet*O_7_-*gtr2*). Overexpression of the *gtr2*^*+*^ gene rescued the gowth defect of *tet*O_7_-*gtr2* cells without ahTet (Fig 1D, *tet*O_7_-*gtr2*). These results suggest that both Lam2 and Gtr2 promote cell growth at least in our conditions. The difference in the phenotypes of Δ*gtr1* cells and Δ*gtr2* cells from the previous reports [19, 34] might be explained by some secondary mutation occurring in the strains used in those reports that restored the capability of cell growth on YES and YPD plates.

**Fig 2:**
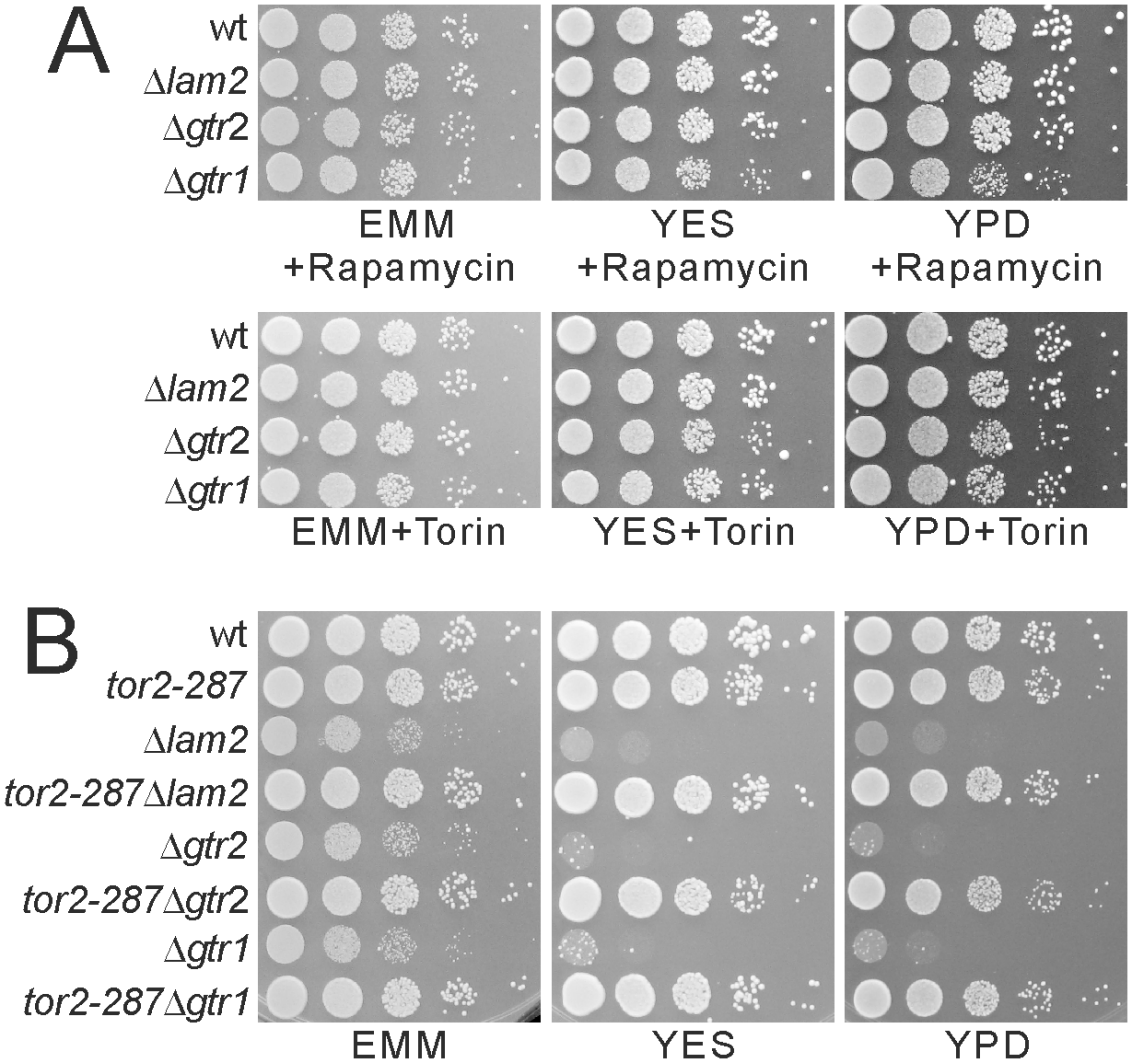
Δ*lam2* cells, Δ*gtr1* cells and Δ*gtr2* cells show growth defects in a TORC1-dependent manner. (A) Growth defects of Δ*lam2* cells, Δ*gtr1* cells and Δ*gtr2* cells were rescued by rapamycin or Torin1. Prototrophic wild-type cells (wt, KP5080), Δ*lam2* cells (KP6578), Δ*gtr1* cells (KP6573) and Δ*gtr2* cells (KP6571) were spotted onto EMM, YES or YPD plates containing 0.2 μg/ml rapamycin or 2 μM Torin1 and incubated as described in Fig 1A. (B) Growth defects of Δ*lam2* cells, Δ*gtr1* cells and Δ*gtr2* cells were rescued by *tor2-287* mutation. Prototrophic wild-type cells (wt, KP5080), *tor2-287* cells (KP6214), Δ*lam2* cells (KP6578), *tor2-287*Δ*lam2* cells (KP6587), Δ*gtr2* cells (KP6571), *tor2-287*Δ*gtr2* cells (KP6583), Δ*gtr1* cells (KP6573) and *tor2-287*Δ*gtr1* cells (KP6672) were spotted and incubated as described in Fig 1A.

To examine whether TORC1 activity is involved in the growth defect of Δ*lam2* cells as well as Δ*gtr1* cells and Δ*gtr2* cells, we treated these cells with TOR inhibitors rapamycin and Torin1 [19, 30, 35, 36]. Both rapamycin (0.2 μg/ml) and Torin1 (2 μM) rescued the growth defects of Δ*lam2* cells, Δ*gtr1* cells and Δ*gtr2* cells (Fig 2A). We generated double mutants of these cells with the *tor2-287* temperature-sensitive mutation [3]. The *tor2*-*287* mutation rescued the growth defect of these cells (Fig 2B). These results show that the growth defects of Δ*lam2* cells, Δ*gtr1* cells and Δ*gtr2* cells are dependent on TORC1 activity.

Our findings so far led us to suspect that Lam2 as well as Gtr1-Gtr2 may negatively regulate TORC1 activity. To examine whether the loss of Npr2 and Npr3, negative regulators for TORC1, phenocopies Δ*lam2* cells, we generated prototrophic cells lacking the *npr2*^+^ or *npr3*^+^ (SPBC543.04) gene (*npr2*::*ura4*^*+*^ or *npr3*::*ura4*^*+*^, respectively), and examined their phenotypes. Similarly to Δ*lam2* cells, the growth of *npr3*::*ura4*^*+*^ cells and *npr2*::*ura4*^*+*^ cells was partially inhibited on EMM plate, and was completely inhibited on both YES and YPD plates (S2A Fig). Overexpression of the *npr3*^*+*^ or *npr2*^*+*^gene completely rescued the growth defect of *npr3*::*ura4*^*+*^ cells or *npr2*::*ura4*^*+*^ cells, respectively (S2B Fig), confirming that the growth defect is due to the loss of Npr3 or Npr2. It was noted, however, that the phenotypes of *npr2*::*ura4*^+^ cells are distinct from those of *npr2*::*KanMX*_*4*_ cells (KP5237) in the previous report [19], which could normally grow on YES and YPD plates and could not growth in the presence of rapamycin. In this study, we obtained *npr2*::*KanMX*_*4*_ progenies (KP6506) from crossing *npr2*::*KanMX*_*4*_ cells used in the previous study (KP5237) with wild-type cells. These progenies did not grow on YES or YPD plates, and this growth defect was rescued by rapamycin (S2A Fig), similarly to *npr2*::*ura4*^+^ cells and Δ*lam2* cells. These phenotypes were rescued by overexpression of the *npr2*^+^ gene (S2B Fig), confirming that these phenotypes are due to the loss of Npr2. Therefore, the phenotypes of *npr2*::*KanMX*_*4*_ cells (KP5237) in the previous report might be irrelevant to the loss of Npr2.

To test whether the growth defect of Δ*npr3* cells and Δ*npr2* cells is due to increased TORC1 activity, we examined whether rapamycin and Torin1 could rescue the growth defect of these cells. Treatment with both rapamycin and Torin1 rescued the growth defects of *npr3*::*ura4*^+^ cells, *npr2*::*ura4*^+^ cells and *npr2*::*KanMX*_*4*_ cells (S3A Fig). Similarly, the *tor2*-*287* mutation rescued the growth defect of these cells (S3B Fig). These results show that the growth defects of Δ*npr3* cells and Δ*npr2* cells are dependent on TORC1 activity, similarly to those of Δ*lam2* cells, Δ*gtr1* cells and Δ*gtr2* cells.

### Δ*lam2* Cells, Δ*gtr1* Cells and Δ*gtr2* Cells Show Abnormal Transcription and Localization of Amino Acid Permeases, Similarly to Δ*npr2* Cells and Δ*npr3* Cells

**Fig 3:**
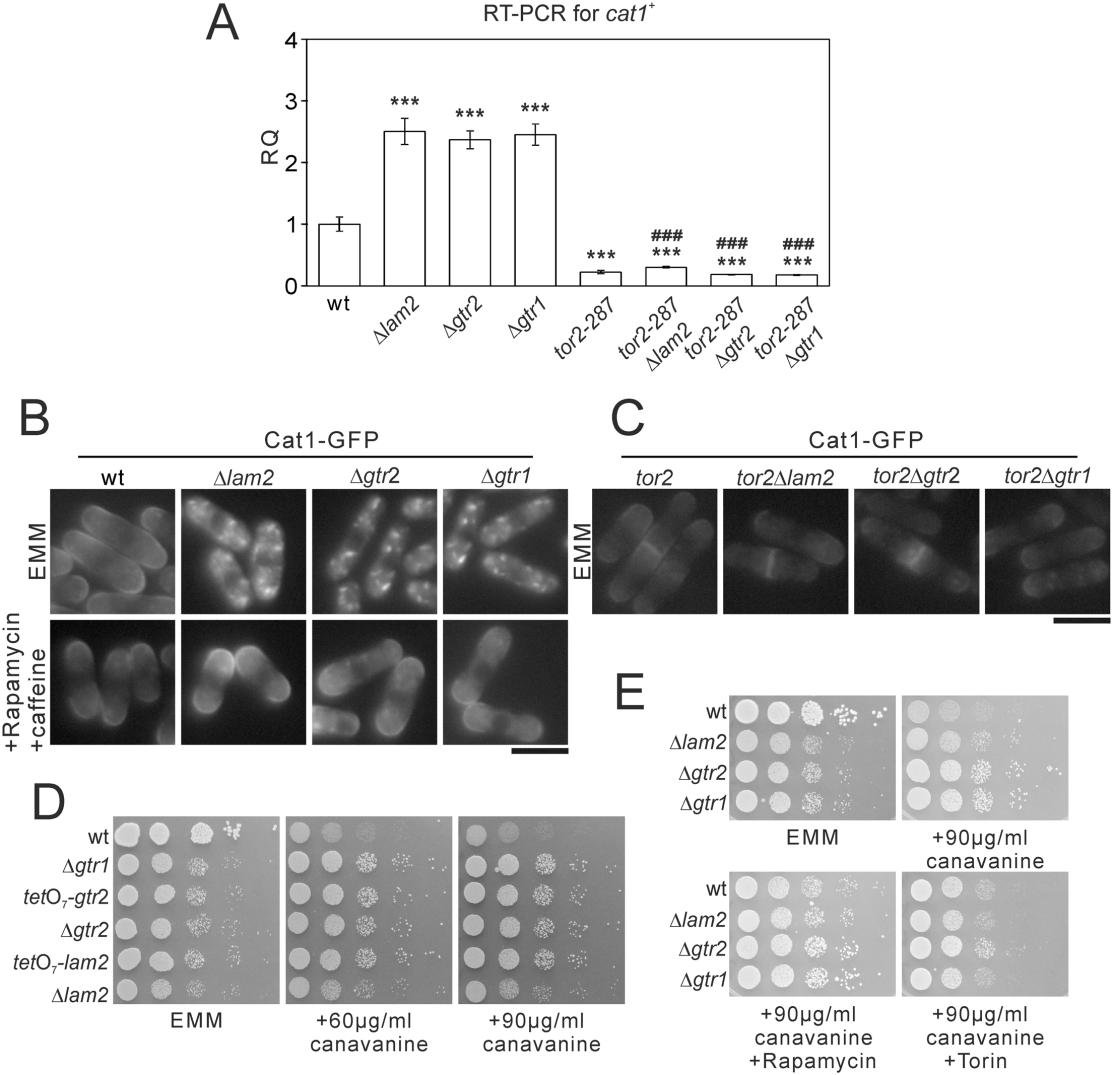
The deficits in Lam2, Gtr1 and Gtr2 increase the expression of *cat1*^+^ and Cat1 internalization in a TORC1-dependent manner. (A) mRNA levels of *cat1*^+^ were increased in Δ*lam2* cells, Δ*gtr2* cells and Δ*gtr1* cells in a TORC1-depedent manner. The cells described in Fig 2B were grown to mid-log phase in EMM medium. Total RNA was extracted from the harvested cells and subjected to quantitative RT-PCR for *cat1*^+^ mRNA. The values were obtained by the comparative CT method in comparison to those of *act1*^+^, and then were normalized to those in wild-type cells (RQ: relative quantity). N = 3 for each group. ***P<0.001 for Turkey’s test following one-way ANOVA for the comparisons with the value of wild-type cells. ^###^*P*<0.001 for Turkey’s test following one-way ANOVA compared with respective single knockout cells. (B) Cat1 internalization in Δ*lam2* cells, Δ*gtr2* cells and Δ*gtr1* cells was abolished upon pharmacological inhibition of TORC1. Wild-type cells (wt, KP5859), Δ*lam2* cells (KP6605), Δ*gtr2* cells (KP6611) and Δ*gtr1* cells (KP6677), expressing Cat1-GFP under its native promoter were grown to mid-log phase in EMM medium. The cells were divided into two portions, one of which was treated with 0.2 μg/ml rapamycin and 10 mM caffeine for 60 min, and the other of which was left untreated. Representative fluorescent images of Cat1-GFP are shown. Scale bar, 10 μm. (C) Cat1 internalization was abolished by simultaneous *tor2-287* mutation. The *tor2-287* cells (KP5955), *tor2-287*Δ*lam2* cells (KP6618), *tor2-287*Δ*gtr2* cells (KP6676) and *tor2-287*Δ*gtr1* cells (KP6679) expressing Cat1-GFP under its native promoter were grown to mid-log phase in EMM medium. Representative fluorescent images of Cat1-GFP are shown. Scale bar, 10 μm. (D) The deficits in Lam2, Gtr1 and Gtr2 caused canavanine resistance. The wild-type cells (wt, KP5080), Δ*gtr1* cells (KP6573), *tet*O_7_-*gtr2* cells (KP6645), Δ*gtr2* cells (KP6571), *tet*O_7_-*lam2* cells (KP6650) and Δ*lam2* cells (KP6578) were spotted onto EMM wihout or with canavanine at 60 or 90 μg/ml. The plates were incubated at 27°C for 4 days without canavanine or for 5 days with canavanine. (E) Δ*lam2* cells, Δ*gtr1* cells and Δ*gtr2* cells showed canavanine resistance in a TORC1-dependent manner. The indicated cells as described in Fig 1A were spotted onto 90 μg/ml canavanine without or with rapamycin or Torin. The plates were incubated at 27°C for 4 days without canavanine or for 5 days with canavanine.

TORC1 regulates gene expression and localization of multiple amino acid permeases in yeast cells. For example, TORC1 activity increases the expression of arginine permease Cat1, inhibits its surface expression, and descreases uptake of canavanine, a toxic arginine analog [19]. Since the phenotypes of Δ*lam2* cells, Δ*gtr1* cells and Δ*gtr2* cells so far are dependent on TORC1 activity, we examined whether these genes also regulate gene expression and localization of Cat1. In Δ*lam2* cells, Δ*gtr1* cells and Δ*gtr2* cells, the mRNA level of *cat1*^+^ was increased compared with wild-type cells (Fig 3A). This increase in *cat1*^+^ expression was abolished in *tor2-287*Δ*lam2* cells, *tor2-287*Δ*gtr1* cells and *tor2-287*Δ*gtr2* cells (Fig 3A), indicating that the increase in *cat1*^+^ expression are dependent on TORC1 activity.

Then we examined the localization of Cat1 using Cat1-GFP expressed under its native promoter. Whereas Cat1-GFP was present at the cell surface in wild-type cells, it was localized to intracellular punctate structures in Δ*lam2* cells, Δ*gtr1* cells and Δ*gtr2* cells (Fig 3B, EMM). These intracellular punctate structures were abolished upon TORC1 inhibition by the combined treatment of rapamycin and caffeine (Fig 3B, Rapamycin + caffeine) or by simultaneous *tor2-287* mutation (Fig 3C). These results indicate that the abnormal localization of Cat1 are dependent on TORC1 activity.

Furthermore, whereas wild-type cells showed growth defect in the presence of canavanine (60 or 90 μg/ml), Δ*lam2* cells, *tet*O_7_-*lam2* cells, Δ*gtr2* cells, *tet*O_7_-*gtr2* cells and Δ*gtr1* cells showed canavanine resistance (Fig 3D). The canavanine resistance of Δ*lam2* cells, Δ*gtr1* cells and Δ*gtr2* cells was partially canceled by rapamycin treatment (Fig 3E, canavanine + Rapamycin). Since the effect of rapamycin was partial, we examined the effect of Torin1. The canavanine resistance of Δ*lam2* cells, Δ*gtr1* cells and Δ*gtr2* cells was completely canceled by Torin1 treatment (Fig 3E, canavanine + Torin). Collectively, the loss of Lam2 as well as Gtr1 and Gtr2 increases gene expression of Cat1 and induces its internalization in a manner dependent on TORC1 activity.

Using *npr3*::*ura4*^+^ cells, *npr2*::*ura4*^+^ cells, and *npr2*::*KanMX*_*4*_ cells, we found that the loss of Npr2 or Npr3 also increases mRNA expression of Cat1 (S4A Fig), induces its internalization (S4B Fig, EMM), and causes canavanine resistance (S4C Fig), similarly to the loss of Lam2 as well as Gtr1 and Gtr2. In Δ*npr2* cells and Δ*npr3* cells, the increase in *cat1*^+^ expression was abolished by simultaneous *tor2-287* mutation (S4A Fig). TORC1 inhibition by rapamycin and caffeine abolished the internalization of Cat1 and increases the signal at the cell surface (S4B Fig, Rapamycin + caffeine). Treatment with rapamycin and caffeine partially canceled, and Torin1 treatment fully canceled, cannavanine resistance in Δ*npr2* cells and Δ*npr3* cells (S4D Fig). Therefore, Δ*npr2* cells and Δ*npr3* cells also show these phenotypes in a manner dependent on TORC1 activity.

Since Cat1 is an arginine permease, we hypothesized that Cat1 internalization in these mutant cells might impede arginine incorporation and consequently causes defective cell growth. Exogenous addition of arginine to the EMM plates enhanced the cell growth of Δ*lam2* cells, Δ*gtr2* cells, Δ*gtr1* cells, Δ*npr3* cells and Δ*npr2* cells as well as wild-type cells, but did not rescue the defective cell growth of these mutant cells to the level of wild-type cells (S5 Fig).

**Fig 4:**
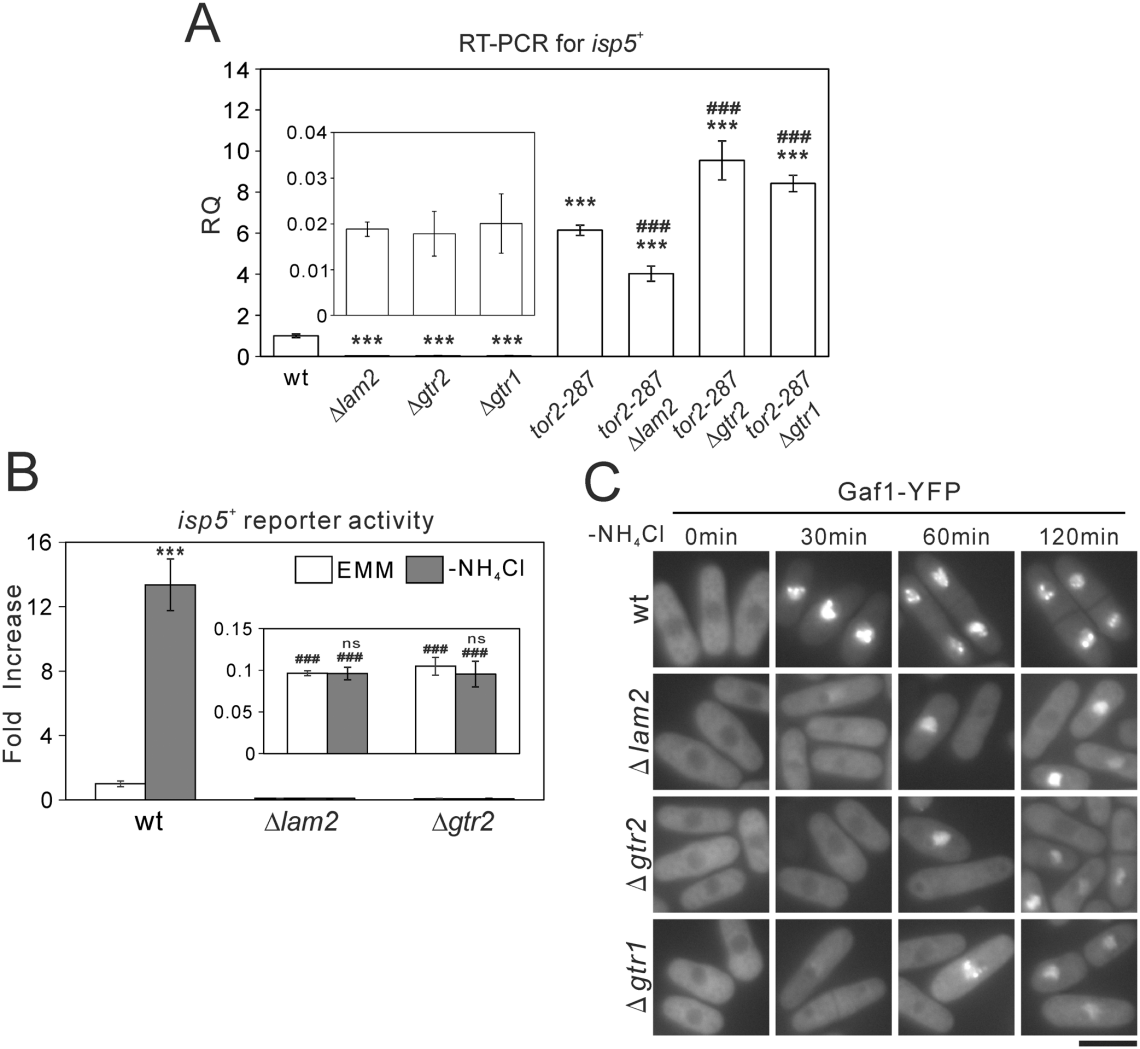
Δ*lam2* cells, Δ*gtr1* cells and Δ*gtr2* cells lack the basal transcription of *isp5*^+^ and its transcriptional activation associated with nuclear Gaf1 localization induced by nitrogen depletion. (A) mRNA levels of *isp5*^+^ were decreased in Δ*lam2* cells, Δ*gtr1* cells and Δ*gtr2* cells in a TORC1-dependent manner. Total RNA from the indicated cells as described in Fig 2B were subjected to quantitative RT-PCR for *isp5*^+^ similarly to Fig 3A. N = 3 for each group. Magnified parts of the graphs are shown in insets. ***P<0.001 for Turkey’s test following one-way ANOVA for the comparisons with the value of wild-type cells. ^###^*P*<0.001 for Turkey’s test following one-way ANOVA compared with respective single knockout cells. (B) Nitrogen depletion-induced *isp5*^+^ transcriptional activation was abolished in Δ*lam2* cells and Δ*gtr2* cells. Wild-type cells (wt, HM123), Δ*lam2* cells (KP6607) and Δ*gtr2* cells (KP6608) harboring the Renilla luciferase reporter plasmid for *isp5*^+^ promoter (pKB8527) were grown to mid-log phase in EMM medium. The medium was replaced by EMM or nitrogen-depleted EMM (-NH_4_Cl), and the cells were subjected to Renilla luciferase reporter assay. Peak values were normalized to that in wild-type cells in the control condition (EMM). N = 3 for each group. Magnified parts of the graphs are shown in insets. ***P<0.001, ns not significant for unpaired *t*-test for the planned comparisons with the same genotype in the control condition. ^###^*P*<0.001 for Turkey’s test following one-way ANOVA compared with wild-type cells in the respective conditions (EMM or -NH_4_Cl). (C) Nuclear localization of Gaf1 upon nitrogen depletion was impaired in Δ*lam2* cells, Δ*gtr2* cells and Δ*gtr1* cells. Wild-type cells (wt, KP6488), Δ*lam2* cells (KP6606), Δ*gtr2* cells (KP6610) and Δ*gtr1* cells (KP6612) expressing Gaf1-YFP under the native Gaf1 promoter were grown to mid-log phase in EMM medium. Fluorescent images of Gaf1-YFP were acquired before (0 min) or after nitrogen depletion (-NH_4_Cl) for the indicated time. Scale bar, 10 μm.

It is also known that TORC1 decreases mRNA levels of *isp5*^+^, another amino acid permease, and that Tor2 inhibition upon nitrogen depletion increases *isp5*^+^ expression [19]. In Δ*lam2* cells, Δ*gtr2* cells and Δ*gtr1* cells, the mRNA level of *isp5*^+^ was decreased, compared with wild-type cells (Fig 4A). This descrease in *isp5*^+^ expression was abolished in *tor2-287*Δ*lam2*, *tor2-287*Δ*gtr2* and *tor2-287*Δ*gtr1* cells (Fig 4A), suggesting the involvement of TORC1 activity. We then examined the transcriptional activity of *isp5*^+^ promoter using Renilla luciferase reporter. In wild-type cells, nitrogen depletion induced the *isp5*^+^ transcriptional activation. By contrast, in Δ*lam2* cells and Δ*gtr2* cells, basal *isp5*^+^ transcriptional activity and its activation induced by nitrogen depletion were abolished (Fig 4B). The mRNA level of *isp5*^+^ (S6A Fig) as well as the basal *isp5*^+^ transcriptional activity and its activation induced by nitrogen depletion as measured by Renilla luciferase reporter (S6B Fig) were also decreased in *npr3*::*ura4*^+^ cells, *npr2*::*ura4*^+^ cells and *npr2*::*KanMX*_*4*_ cells. The descrease in *isp5*^+^ mRNA was abolished in *tor2-287*Δ*npr2* and *tor2-287*Δ*npr3* cells (S6A Fig), suggesting the involvement of TORC1 activity as well.

In fission yeast, TORC1 inhibition under nitrogen depletion induces nuclear localization of a GATA transcription factor Gaf1, and Gaf1 is critical for the basal *isp5*^+^ transcriptional activity and its activation induced by nitrogen depletion [32]. These findings prompted us to examine the localization of Gaf1 using Gaf1-YFP expressed under its native promoter. In wild-type cells cultured in the EMM medium, Gaf1-YFP was localized to the cytosol (Fig 4C, wt, 0 min). Nitrogen depletion induced robust nuclear localization of Gaf1 from the cytosol within 30 min, and this nuclear localization was sustained at least up to 120 min (Fig 4C, wt). In Δ*lam2* cells, Δ*gtr2* cells and Δ*gtr1* cells, the nuclear localization of Gaf1-YFP was not observed within 30 min after nitrogen depletion. Although a subset of these mutant cells showed the nuclear localization of Gaf1-YFP at 60 and 120 min, its nuclear localization appeared to be partial, compared with wild-type cells (Fig 4C). Using *npr3*::*ura4*^+^ cells, *npr2*::*ura4*^+^ cells and *npr2*::*KanMX*_*4*_ cells, we found that the loss of Npr2 or Npr3 also impairs the nuclear localization of Gaf1-YFP induced by nitrogen depletion (S6C Fig), similarly to the loss of Lam2.

### Δ*lam2* Cells, Δ*gtr1* Cells and Δ*gtr2* Cells Show Elevated TORC1 Activity under Nitrogen Depletion, Similarly to Δ*npr2* Cells and Δ*npr3* Cells

**Fig 5:**
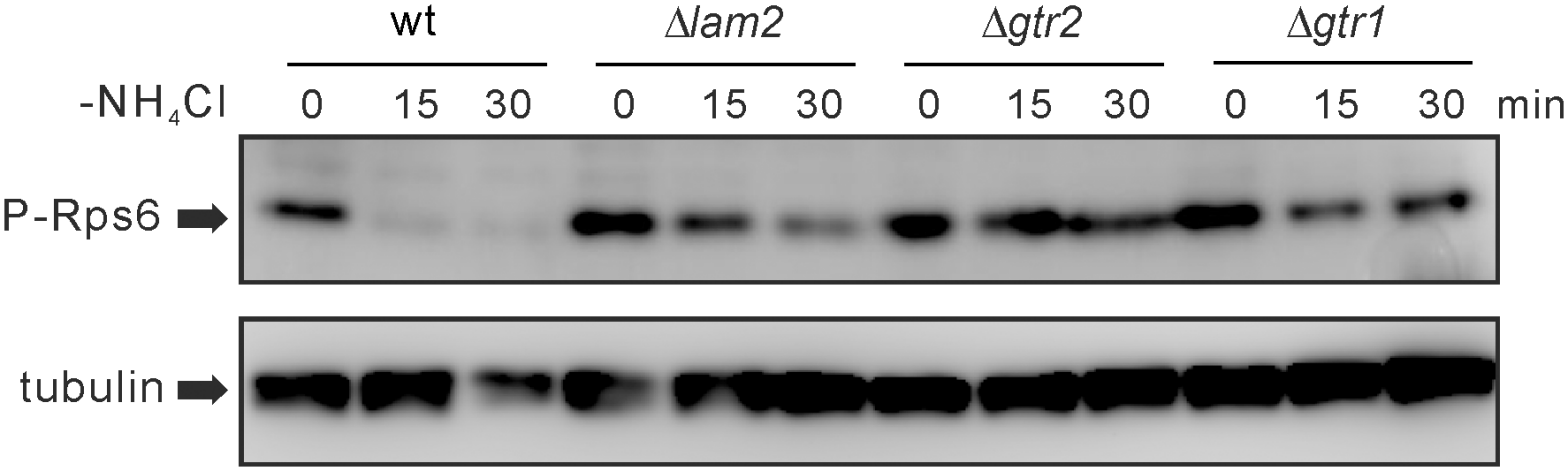
Nitrogen depletion-induced dephosphorylation of Rps6 is inhibited in Δ*lam2* cells, Δ*gtr1* cells and Δ*gtr2* cells. Prototrophic wild-type cells (wt, KP5080), Δ*lam2* cells (KP6578), Δ*gtr2* cells (KP6571) and Δ*gtr1* cells (KP6573) were grown to mid-log phase in EMM medium. The cells were harvested before (0 min) or after nitrogen depletion (-NH_4_Cl) for 15 or 30 min, and the proteins were extracted from these cells. The cell lysates were subjected to immunoblot analysis for Rps6 phosphorylation (P-Rps6) as a readout for TORC1 activity. α-Tubulin was detected as an internal control.

Our findings so far show that Δ*lam2* cells, Δ*gtr1* cells and Δ*gtr2* cells phenocopy Δ*npr2* cells and Δ*npr3* cells in a TORC1-dependent manner. To examine how these molecules regulate TORC1 activity, we examined Rps6 phosphorylation in these cells as a readout of TORC1 activity [19, 30, 37]. In nitrogen-rich conditions, Rps6 phosphorylation appeared to be normal in these mutant cells, compared with wild-type cells (Fig 5 and S7 Fig). In wild-type cells, nitrogen depletion suppressed Rps6 phosphorylation within 15 min after nitrogen depletion (Fig 5). By contrast, Δ*lam2* cells, Δ*gtr1* cells and Δ*gtr2* cells showed increased Rps6 phosphorylation under nitrogen depletion, compared with wild-type cells, especially at 15 min (Fig 5). Δ*npr2* cells and Δ*npr3* cells also showed increased Rps6 phosphorylation (S7 Fig), similarly to the loss of Lam2, Gtr1 or Gtr2. These results suggest that Lam2 as well as Gtr1 and Gtr2 functions as a negative regulator for TORC1, similarly to Npr2 and Npr3, at least under nitrogen depletion.

### Genetic interactions between Lam2, Gtr1-Gtr2 and Npr2-Npr3

**Fig 6:**
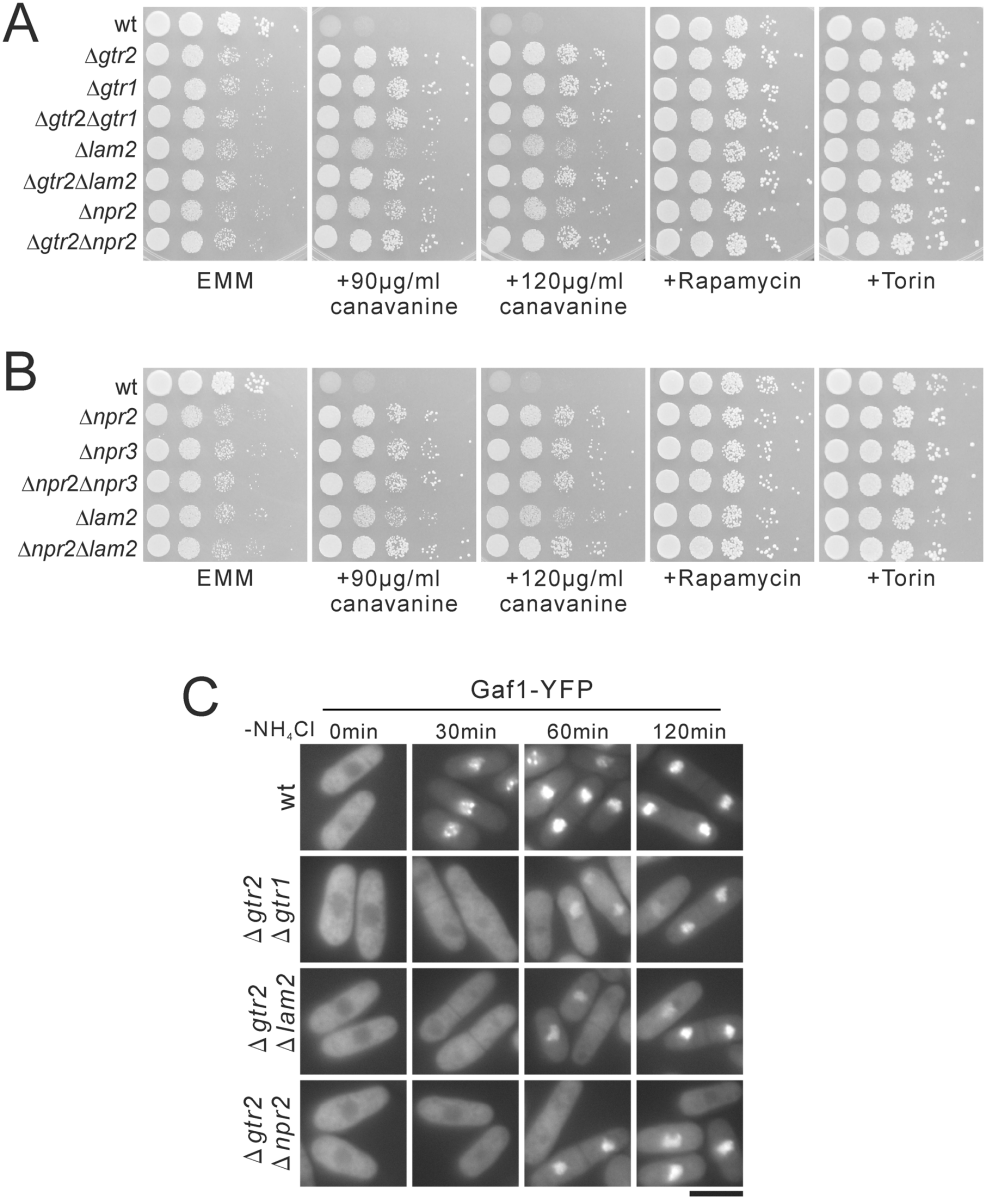
The double knockout cells of *lam2*, *gtr2* and *npr2* lack synthetic effects in growth defect, canavanine resistance, or nuclear localization of Gaf1 induced by nitrogen depletion. (A, B) Δ*gtr2*Δ*gtr1* cells, Δ*gtr2*Δ*lam2* cells, Δ*gtr2*Δ*npr2* cells, Δ*npr2*Δ*npr3* cells, Δ*npr2*Δ*lam2* cells lacked synthetic effects in growth defect. The indicated prototrophic wild-type cells (wt, KP5080), Δ*gtr2* cells (KP6571), Δ*gtr1* cells (KP6573), Δ*gtr2*Δ*gtr1* cells (KP6684), Δ*lam2* cells (KP6578), Δ*gtr2*Δ*lam2* cells (KP6590), Δ*npr2* cells (KP6506), Δ*gtr2*Δ*npr2* cells (KP6588), Δ*npr3* cells (KP6552), Δ*npr2*Δ*npr3* cells (KP6683) and Δ*npr2*Δ*lam2* cells (KP6682) were spotted onto the indicated plates, and then incubated at 27°C on EMM plates for 4 days, on EMM plates containing canavanine for 5 days, and on EMM plates containg rapamycin or Torin for 4 days. (C) Δ*gtr2*Δ*gtr1* cells, Δ*gtr2*Δ*lam2* cells and Δ*gtr2*Δ*npr2* cells lacked synthetic effects in nuclear localization of Gaf1 induced by nitrogen depletion. Wild-type cells (wt, KP6488), Δ*gtr2*Δ*gtr1* cells (KP6687), Δ*gtr2*Δ*lam2* cells (KP6685) and Δ*gtr2*Δ*npr2* cells (KP6686) expressing Gaf1-YFP under the native Gaf1 promoter were grown to mid-log phase in EMM medium. Fluorescent images of Gaf1-YFP were then acquired before (0 min) or after nitrogen depletion (-NH_4_Cl) for the indicated time. Scale bar, 10 μm.

To examine the relationship among Lam2, Gtr1-Gtr2 and Npr2-Npr3, we examined whether the double mutants of these molecules showed synthetic effects. The growth defects of Δ*gtr2*Δ*gtr1* cells, Δ*gtr2*Δ*lam2* cells, Δ*gtr2*Δ*npr2* cells, Δ*npr2*Δ*npr3* cells and Δ*npr2*Δ*lam2* cells on EMM plates were similar to those of the respective single mutant cells (Fig 6A and 6B). No apparent synthetic effects of these double mutant cells were observed in either canavanine resistance or the rescue of growth defect by TOR inhibitors (Fig 6A and 6B). We also examined whether the double mutants showed synthetic effects in nuclear localization of Gaf1-YFP induced by nitrogen depletion. Thus, Δ*gtr2*Δ*gtr1* cells, Δ*gtr2*Δ*lam2* cells and Δ*gtr2*Δ*npr2* cells showed the lack of nuclear localization of Gaf1-YFP at 30 min after nitrogen depletion (Fig 6C), similarly to the respective single knockout cells (Fig 4C). At 60 and 120 min, a subset of these double mutant cells showed nuclear localization of Gaf1-YFP, but the intensity of nuclear Gaf1-YFP appeared to be lower than that in wild-type cells (Fig 6C), similarly to the respective single knockout cells (Fig 4C). The lack of synthetic effects of these double mutant cells suggest that Lam2 and Gtr1-Gtr2 function in the same pathway as Npr2-Npr3.

### Lam2 and Npr2-Npr3 Form a Physical Complex with Gtr1 and Similarly Promote the Vacuolar Localization of Gtr1-Gtr2

**Fig 7:**
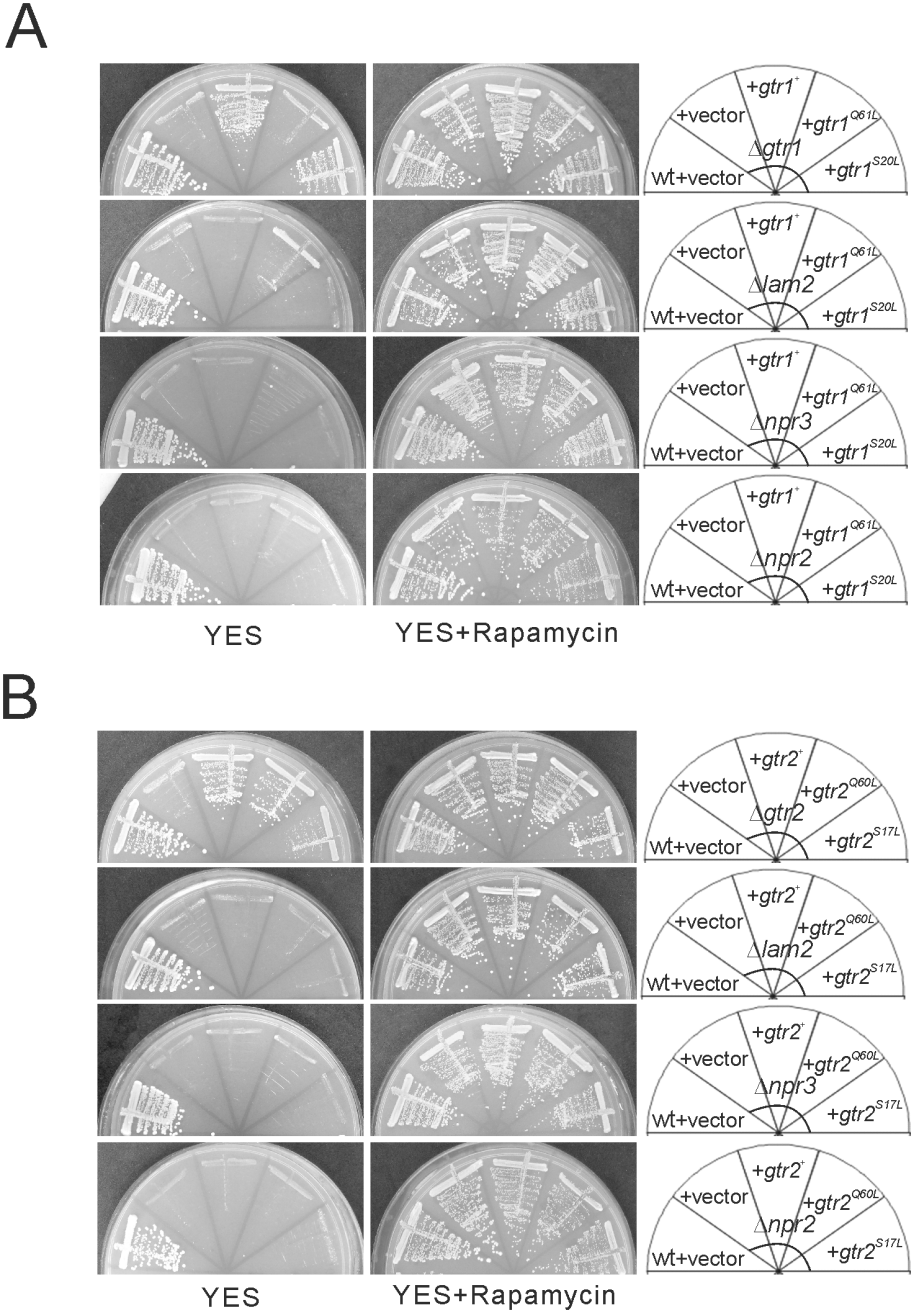
GDP-bound Gtr1 and GTP-bound Gtr2 promote the cell growth in a manner dependent on Lam2, Npr2 and Npr3. (A) The leucine auxotrophic Δ*gtr1* cells (KP6688), Δ*lam2* cells (KP6607), Δ*npr3* cells (KP6551) and Δ*npr2* cells (KP6586) transformed with the control vector (+ vector, pKB2437) or the plasmid expressing Gtr1 (pKB8676), Gtr1^Q61L^ (pKB8910) or Gtr1^S20L^ (pKB8911) as well as wild-type cells (wt, HM123) transformed with the control vector (pKB2437) were streaked onto YES plates without or with rapamycin. The plates were incubated at 27°C for 4 days. (B) The leucine auxotrophic Δ*gtr2* cells (KP6608), Δ*lam2* cells (KP6607), Δ*npr3* cells (KP6551) and Δ*npr2* cells (KP6586) transformed with the control vector (+ vector, pKB2437) or the plasmid expressing Gtr2 (pKB8634), Gtr2^Q60L^ (pKB8905) or Gtr2^S17L^ (pKB8904) as well as wild-type cells (wt, HM123) transformed with the control vector (pKB2437) were streaked onto YES plates without or with rapamycin. The plates were incubated at 27°C for 4 days.

To characterize the relationship between Gtr1-Gtr2, Lam2 and Npr2-Npr3, we examined whether Gtr1 and Gtr2 as well as their GTP-locked and GDP-locked mutants could rescue the phenotypes of Δ*gtr1* cells, Δ*gtr2* cells, Δ*lam2* cells, Δ*npr2* cells and Δ*npr3* cells. Both wild-type Gtr1 and GDP-locked Gtr1^S20L^, but not GTP-locked Gtr1^Q61L^, rescued the defective growth in Δ*gtr1* cells (Fig 7A). In contrast, wild-type Gtr2 and GTP-locked Gtr2^Q60L^, but not GDP-locked Gtr2^S17L^, rescued the the defective growth in Δ*gtr2* cells (Fig 7B). These findings suggest that GDP-bound Gtr1 and GTP-bound Gtr2 are necessary for the cell growth on YES plates. However, neither wild-type Gtr1, GTP-locked Gtr1^Q61L^ nor GDP-locked Gtr1^S20L^ rescued the defective growth in Δ*lam2* cells, Δ*npr3* cells and Δ*npr2* cells (Fig 7A). Furthermore, neither wild-type Gtr2, GTP-locked Gtr2^Q60L^ nor GDP-locked Gtr2^S17L^ rescued the defective growth in these mutant cells (Fig 7B). Overexpression of wild-type Gtr1 and Gtr2 as well as their GTP-locked and GDP-locked mutants did not affect the cell growth of these mutant cells in the presence of rapamycin, either (Fig 7A and 7B). Therefore, these findings suggest that the defective growth of Δ*lam2* cells, Δ*npr2* cells and Δ*npr3* cells are not due to altered nucleotide-bound states of Gtr1 or Gtr2.

**Fig 8:**
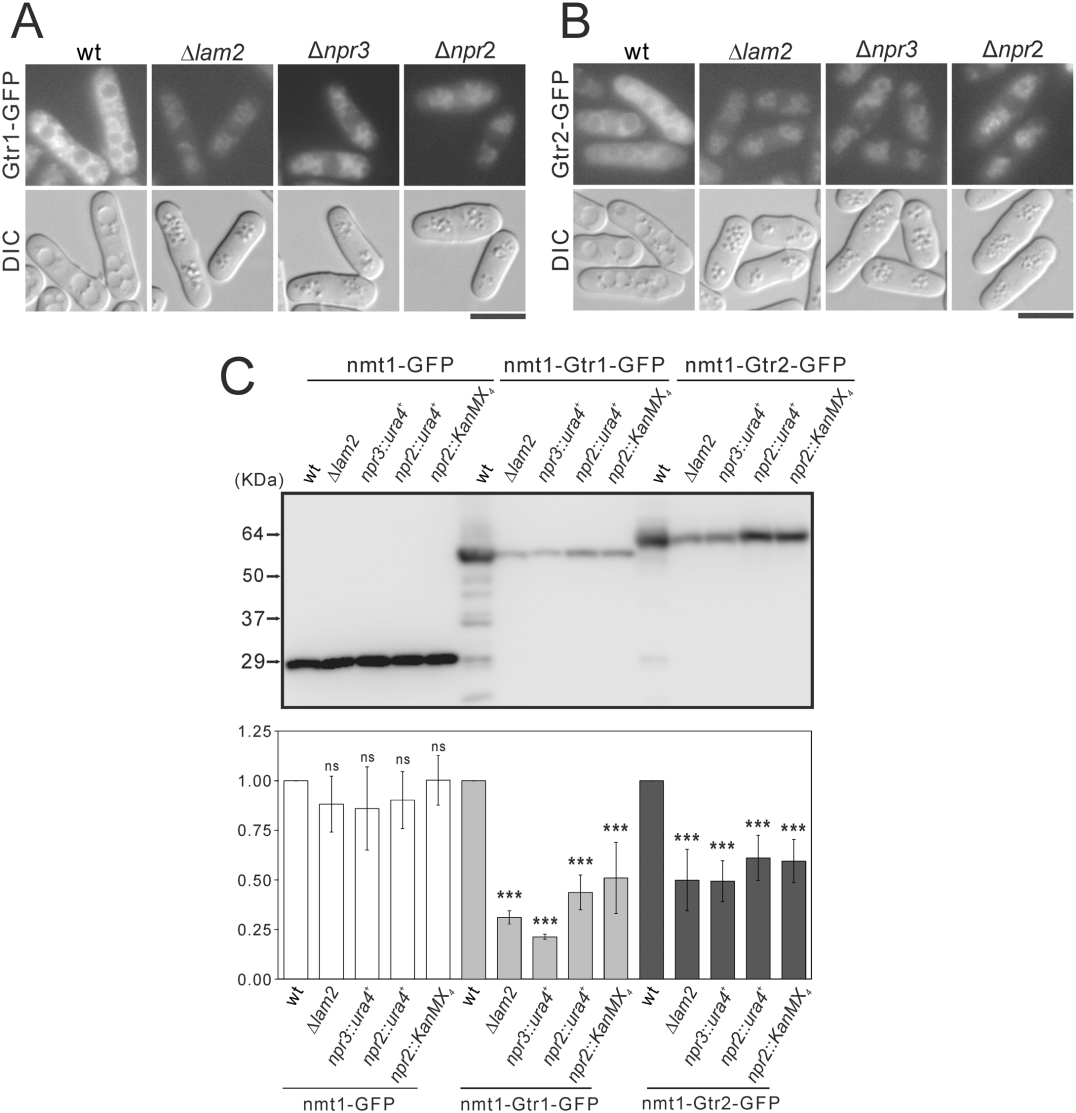
Lam2, Npr3 and Npr2 are necessary for maintaining the vacuolar localization and the protein levels of Gtr1 and Gtr2. (A, B) Intracellular localization of Gtr1-GFP (A) or Gtr2-GFP (B) in wild-type (wt) cells, Δ*lam2* cells, Δ*npr3* cells or Δ*npr2* cells are shown. The leucine auxotrophic wild-type (wt, HM123), Δ*lam2* cells (KP6607), Δ*npr3* cells (KP6551) and Δ*npr2* cells (KP6586) transformed with the plasmids expressing Gtr1-GFP (pKB8702) (A) or Gtr2-GFP (pKB9133) (B) under *nmt1* promoter were grown to early log phase in EMM medium at 27°C, and then were shifted to YES medium overnight. The fluorescent and DIC images were then acquired after the cells were treated with distilled water for 30 min. Scale bar, 10 μm. (C) The protein levels of Gtr1-GFP and Gtr2-GFP in wild-type (wt) cells, Δ*lam2* cells, Δ*npr3* cells and Δ*npr2* cells. The leucine auxotrophic wild-type (wt, HM123), Δ*lam2* cells (KP6607), *npr3*::*ura4*^+^ cells (KP6551), *npr2*::*ura4*^+^ cells (KP6586) and *npr2*::*KanMX*_*4*_ cells (KP6585) transformed the plasmids expressing, GFP (pKB2728), Gtr1-GFP (pKB8702) or Gtr2-GFP (pKB9133) under *nmt1* promoter were grown to early log phase in EMM medium without thiamine at 27°C for 20 h. Proteins were extracted and subjected to SDS-PAGE and immunoblot analyses with anti-GFP antibodies. A representative image of immunoblot analysis is shown in the upper panel. Signal intensities of the corresponding bands were quantified and normalized to the values of wild-type cells. The resultant normalized intensities were averaged across three independent blots, and are shown in the lower panel. ***P<0.001 for Turkey’s test following one-way ANOVA for the comparisons with the value of wild-type cells.

Then we investigated the possibility that Lam2, Npr2 and Npr3 might fuction as a tether for Gtr1 and Gtr2. First, we observed the localization of Gtr1-GFP expressed under *nmt1* promoter. In wild-type cells, hypotonic stress induced a fusion of vacuoles, and Gtr1-GFP and Gtr2-GFP were localized at vacuolar membranes. In Δ*lam2* cells, Δ*npr2* cells and Δ*npr3* cells, vacuoles were smaller, and the intensities of Gtr1-GFP and Gtr2-GFP at vacuolar membranes were decreased, compared with wild-type cells (Fig 8A and 8B). These findings suggest that Lam2 and Npr2-Npr3 similarly promote the vacuolar localization of Gtr1 and Gtr2 as a tether for Gtr1-Gtr2 at vacuolar membranes. In immunoblotting, the protein levels of Gtr1-GFP and Gtr2-GFP, but not of GFP control vector, were reduced in Δ*lam2* cells, Δ*npr3* cells and Δ*npr2* cells, compared with wild-type cells (Fig 8C), suggesting that Lam2 and Npr2-Npr3 maintain the protein levels of Gtr1 and Gtr2.

**Fig 9:**
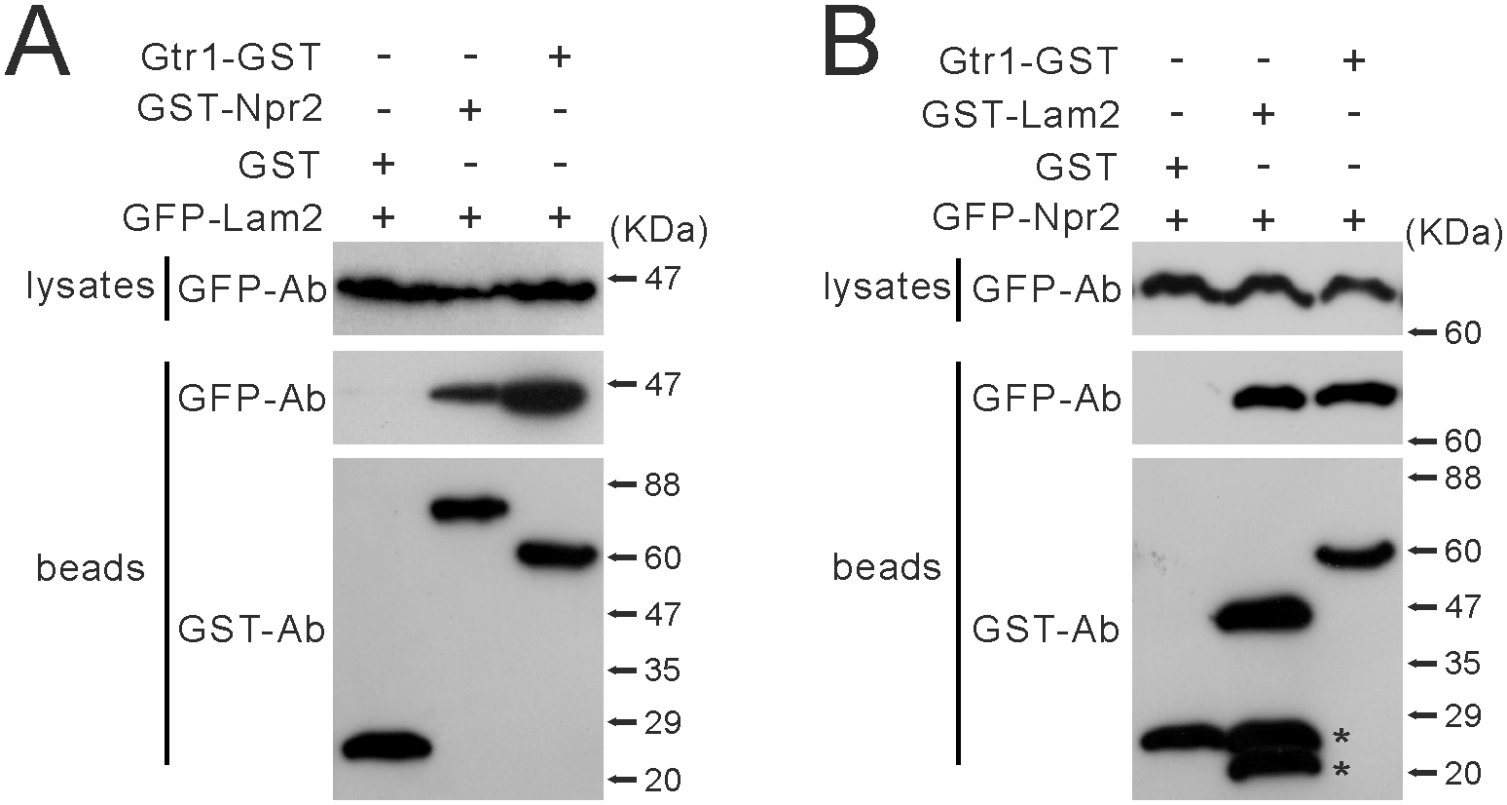
Lam2 physically interacts with Npr2 and Gtr1. (A) GST-Npr2 and Gtr1-GST were co-precipitated with GFP-Lam2. GFP-Lam2-integrated cells (KP6192) expressing Gtr1-GST (pKB8676), GST-Npr2 (pKB8567) or GST (pKB2437) under the *nmt1* promoter were grown in EMM medium without thiamine for 20 hr. Gtr1-GST, GST-Npr2 and GST were precipitated from the cell lysates by glutathione beads. The precipitated proteins were subjected to immunoblot analyses using anti-GFP and anti-GST antibodies (Ab). (B) GST-Lam2 and Gtr1-GST were co-precipitated with GFP-Npr2. GFP-Npr2-integrated cells (KP6283) expressing Gtr1-GST (pKB8676), GST-Lam2 (pKB8795) or GST (pKB2437) were grown in EMM medium without thiamine for 20 hr. Gtr1-GST, GST-Lam2 and GST were precipitated from the cell lysates by glutathione beads, and the precipitated proteins were subjected to immunoblot analyses using anti-GFP and anti-GST antibodies (Ab). The asterisks indicate unexpected bands may represent degradation products.

Since our findings suggest that Npr2 and Npr3 function similarly to Lam2, we investigated whether Lam2 could form a physical complex with Npr2. We expressed GST (glutathione S-transferase), Gtr1-GST or GST-Npr2 together with GFP-Lam2, and subjected these cells to the co-precipitation assay with glutathione sepharose 4B beads. GST-Npr2 and Gtr1-GST, but not GST, were co-precipitated with GFP-Lam2 (Fig 9A). On the other hand, we expressed GST, GST-Lam2 or Gtr1-GST together with GFP-Npr2, and subjected these cells to the co-precipitation assay with glutathione beads. GST-Lam2 and Gtr1-GST, but not GST, were co-precipitated with GFP-Npr2 (Fig 9B). These findings suggest that Lam2 forms a physical complex with Gtr1 and Npr2.

## Discussion

In fission yeast, whether Lam2 functions as a component of a GEF or tether for Gtr1 remains unknown. Whether Npr2-Npr3 functions only as a component of a GAP for Gtr1 is also elusive. In this study, we found that the loss of Lam2 and Gtr1-Gtr2 phenocopies the loss of Npr2-Npr3: The loss of Lam2, Gtr1, Gtr2, Npr2 or Npr3 similarly causes growth defect, induces transcription of *cat1*^+^ and Cat1 internalization, and decreases the basal transcription of *isp5*^+^ and its activation associated with nuclear Gaf1 localization induced by nitrogen depletion. All these phenotypes are rescued by pharmacological or genetic inhibition of TORC1, and the loss of any of these genes increases TORC1 activity at least after nitrogen depletion. These findings suggest that Lam2, Gtr1-Gtr2 and Npr2-Npr3 all mediate the above phenotypes through suppressing TORC1 activity. Consistently, the overexpression of GDP-bound Gtr1 and GTP-bound Gtr2, which suppress TORC1 activity in combination in budding yeast [14], rescues the phenotypes in the cells lacking Gtr1 and Gtr2, respectively, but not in those lacking Lam2, Npr2 or Npr3. Furthermore, Lam2 and Npr2-Npr3 maintain the vacuolar localization of Gtr1 and Gtr2, and form a physical complex with Gtr1. These findings suggest that Lam2 and Npr2-Npr3 function together as a tether for GDP-bound Gtr1 to the vacuolar membrane, thereby negatively regulating TORC1 activity in fission yeast.

Studies in budding yeast have shown that GTP-bound Gtr1 preferentially binds to and activates TORC1 [18]. Our findings suggest that GDP-bound Gtr1 plays an active role in suppressing TORC1 activity to promote cell growth. Indeed it has been reported that GDP-bound Rag GTPases recruit TSC2 to inactivate mTORC1 upon amino acid removal in mammalian cells [38]. Therefore, the loss of Gtr1 could inhibit or activate TORC1, depending on the GTP/GDP ratio of Gtr1 in a given experimental condition. Notably, the loss of Gtr1-Gtr2 as well as the loss of Lam2 or Npr2-Npr3 increases Rps6 phosphorylation only after nitrogen depletion, and this increase was not evident in the nitrogen-rich condition. Since Rps6 is a ribosomal protein that is phosphorylated by Psk1, a direct substrate of TORC1 [37], Rps6 phosphorylation could not be sensitive enough to detect local changes in TORC1 activity at the vacuolar membrane.

LAMTOR2 is a component of Ragulator, which is thought to function as a GEF for RagA and RagB in mammalian cells [17]. If Lam2 might function only as a component of GEF for Gtr1, it would be expected that the loss of Lam2 decreases the GTP-bound Gtr1 and consequently suppresses TORC1 activity in fission yeast. This expectation is contrary to our finding that the loss of Lam2 disinhibits TORC1 activity. Since studies in yeast have revealed Vam6 as a GEF for Gtr1 [14, 18], the Ragulator complex may play another role. Our findings favor the possibility that Lam2 functions as a component of tether for Gtr1, because the loss of Lam2 attenuates the localization of Gtr1 and Gtr2 on the vacuolar membrane. Since GDP-bound Gtr1 is necessary to promote cell growth through suppressing TORC1 in our experimental condition, it is plausible that Lam2 tethers GDP-bound Gtr1 at the vacuolar membrane, thereby suppressing TORC1 activity. However, whether GDP-bound Gtr1 can be localized on the vacular membrane similarly to GTP-bound Gtr1 remains to be proven.

In the SEACIT complex, Iml1 has a domain for GAP activity for Gtr1 [10], and whether Npr2-Npr3 affects this GAP activity remains unknown. We found that Lam2 forms a complex with Npr2 at least with overexpression of these proteins, and that the loss of Npr2-Npr3 diminishes the vacuolar localization of Gtr1, similarly to the loss of Lam2. In addition, our findings suggest that GDP-bound Gtr1 promotes cell growth in a manner dependent on Lam2 and Npr2-Npr3. Therefore, Npr2-Npr3 together with Lam2 could also tether GDP-bound Gtr1 on the vacuolar membrane. In budding yeast, Npr2 and Npr3 are localized to the vacuolar membrane [8]. Since the Ragulator complex is anchored to the lysosomal membrane through its component LAMTOR1 [7], Lam2 could promote the vacuolar localization of Npr2-Npr3 through the physical interaction.

In conclusion, this study suggests a role of Lam2 and Npr2-Npr3 as a tether for Gtr1 to the vacuolar membrane for multiple cellular functions, and thus proposes a novel role of Npr2-Npr3 beyond a GAP for Gtr1 at least in fission yeast. Whether this finding is exploitable to understanding the functions of regulators for Rag GTPases, such as the Ragulator and GATOR complexes, in mammalian cells warrants future investigations.

## Supporting Information

S1 Fig Graphical models for upstream regulators of TORC1 in mammals and yeasts. (TIF)

S2 Fig The deficits in Npr3 and Npr2 cause growth defect. (TIF)

S3 Fig Δ*npr3* cells and Δ*npr2* cells show growth defects in a TORC1-dependent manner. (TIF)

S4 Fig The deficits in Npr3 and Npr2 increase the expression of *cat1*^+^ and Cat1 internalization in a TORC1-dependent manner. (TIF)

S5 Fig Exogenous addition of arginine did not rescue the defective cell growth of Δ*lam2* cells, Δ*gtr2* cells, Δ*gtr1* cells, Δ*npr3* cells and Δ*npr2* cells to the level of wild-type cells. (TIF)

S6 Fig Δ*npr3* cells and Δ*npr2* cells lack the basal transcription of *isp5*^+^ and its transcriptional activation associated with nuclear Gaf1 localization induced by nitrogen depletion. (TIF)

S7 Fig Nitrogen depletion-induced dephosphorylation of Rps6 is inhibited in Δ*npr3* cells and Δ*npr2* cells. (TIF)

S1 Table Fission yeast strains used in this study. (DOCX)
