# Supplementary figures and images for: The Loss of Lam2 and Npr2-Npr3 Diminishes the Vacuolar Localization of Gtr1-Gtr2 and Disinhibits TORC1 Activity in Fission Yeast
Source: PLoS One. 2016 May 26;11(5):e0156239. doi: 10.1371/journal.pone.0156239 (PMC4881991; doi:10.1371/journal.pone.0156239)

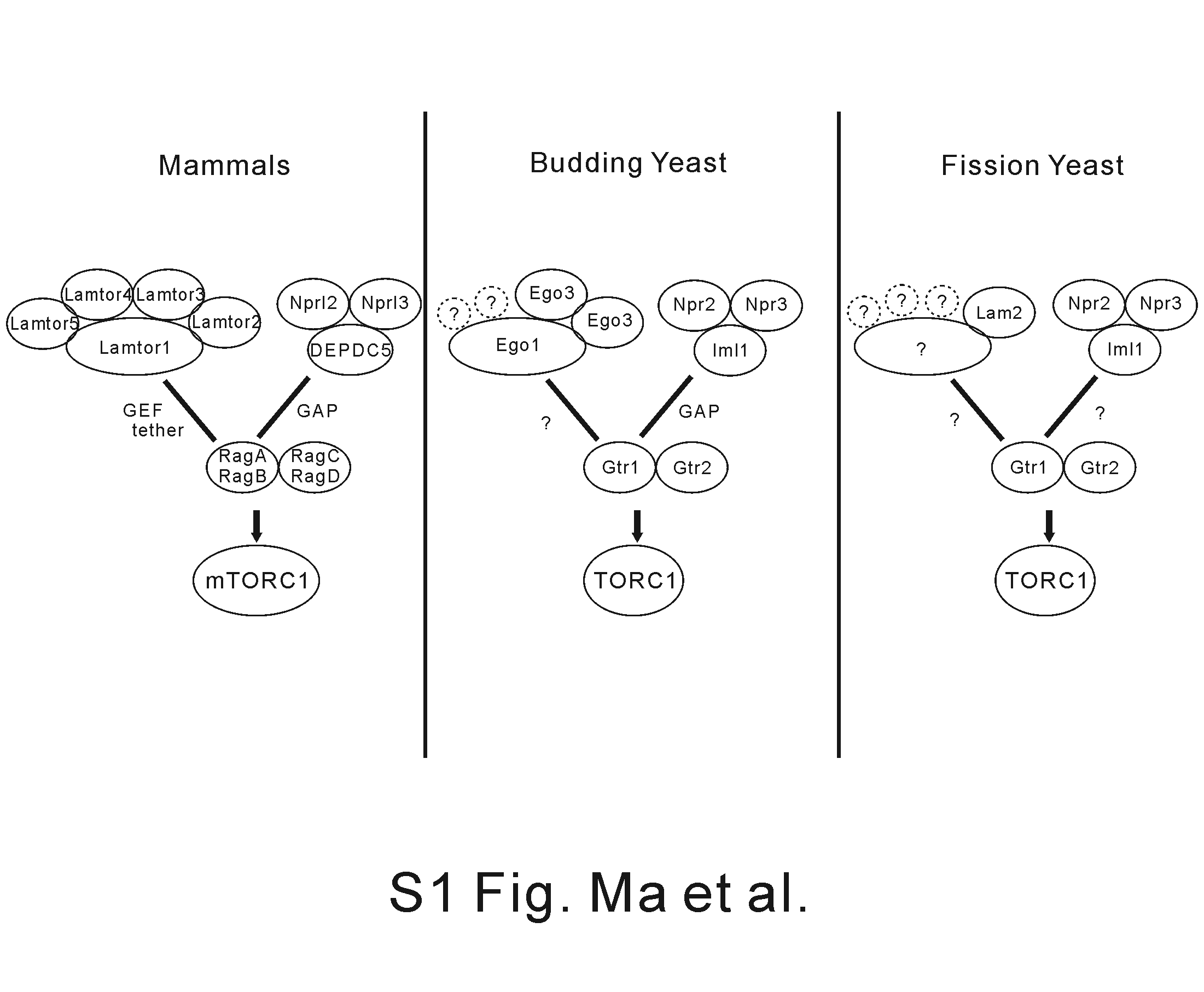

Supplement: S1 Fig — (TIF) [file pone.0156239.s001.tif]

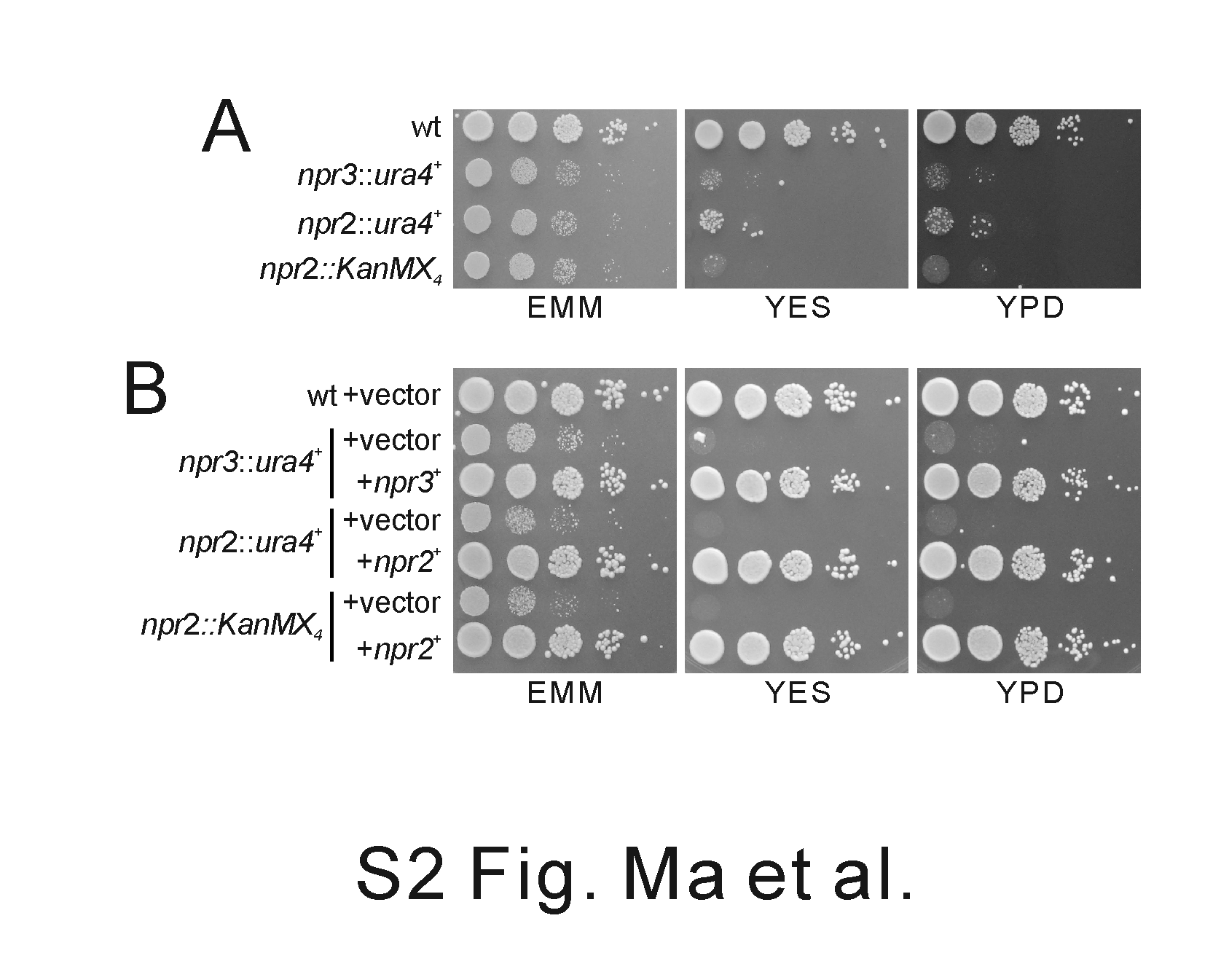

Supplement: S2 Fig — (TIF) [file pone.0156239.s002.tif]

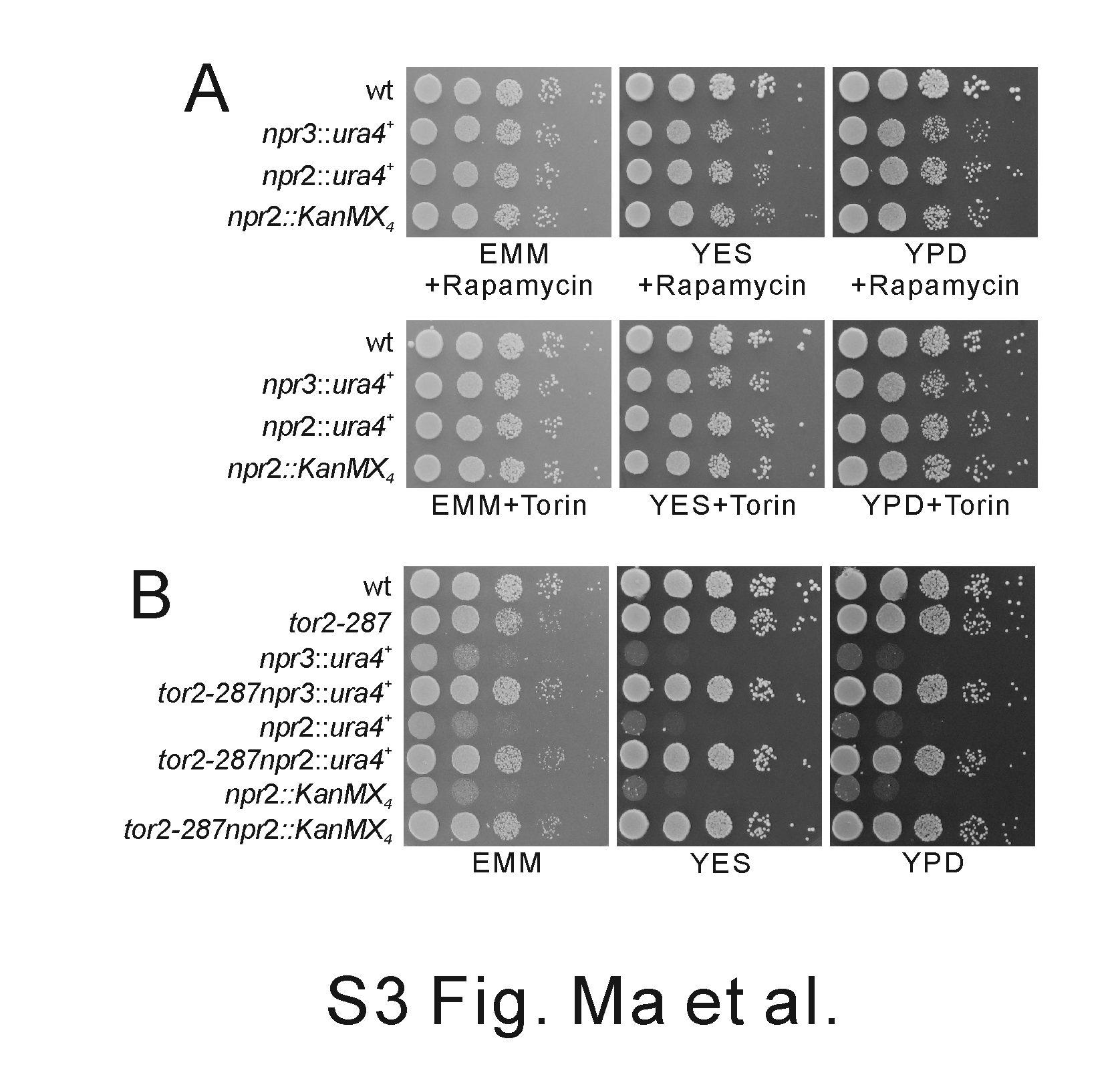

Supplement: S3 Fig — (TIF) [file pone.0156239.s003.tif]

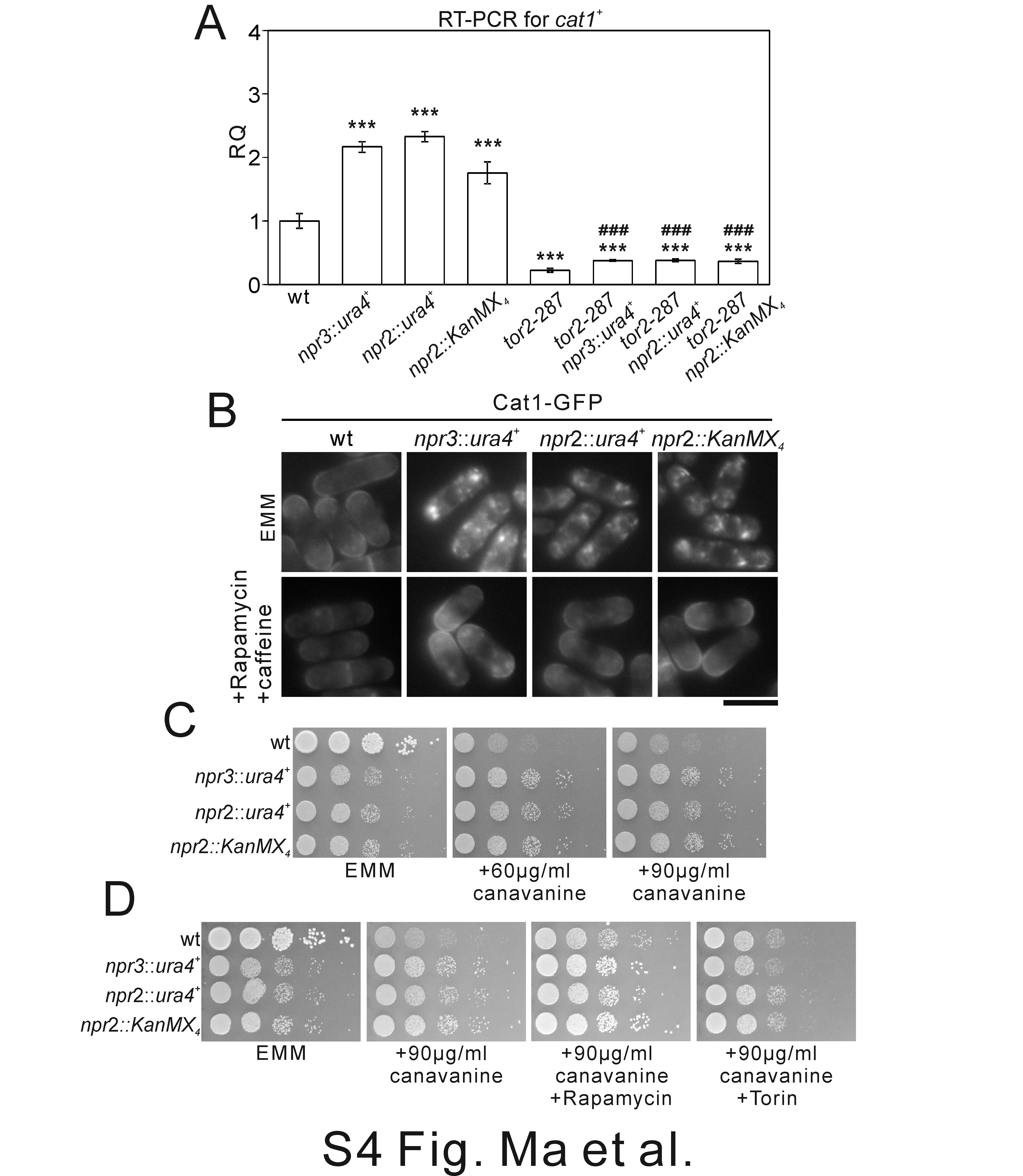

Supplement: S4 Fig — (TIF) [file pone.0156239.s004.tif]

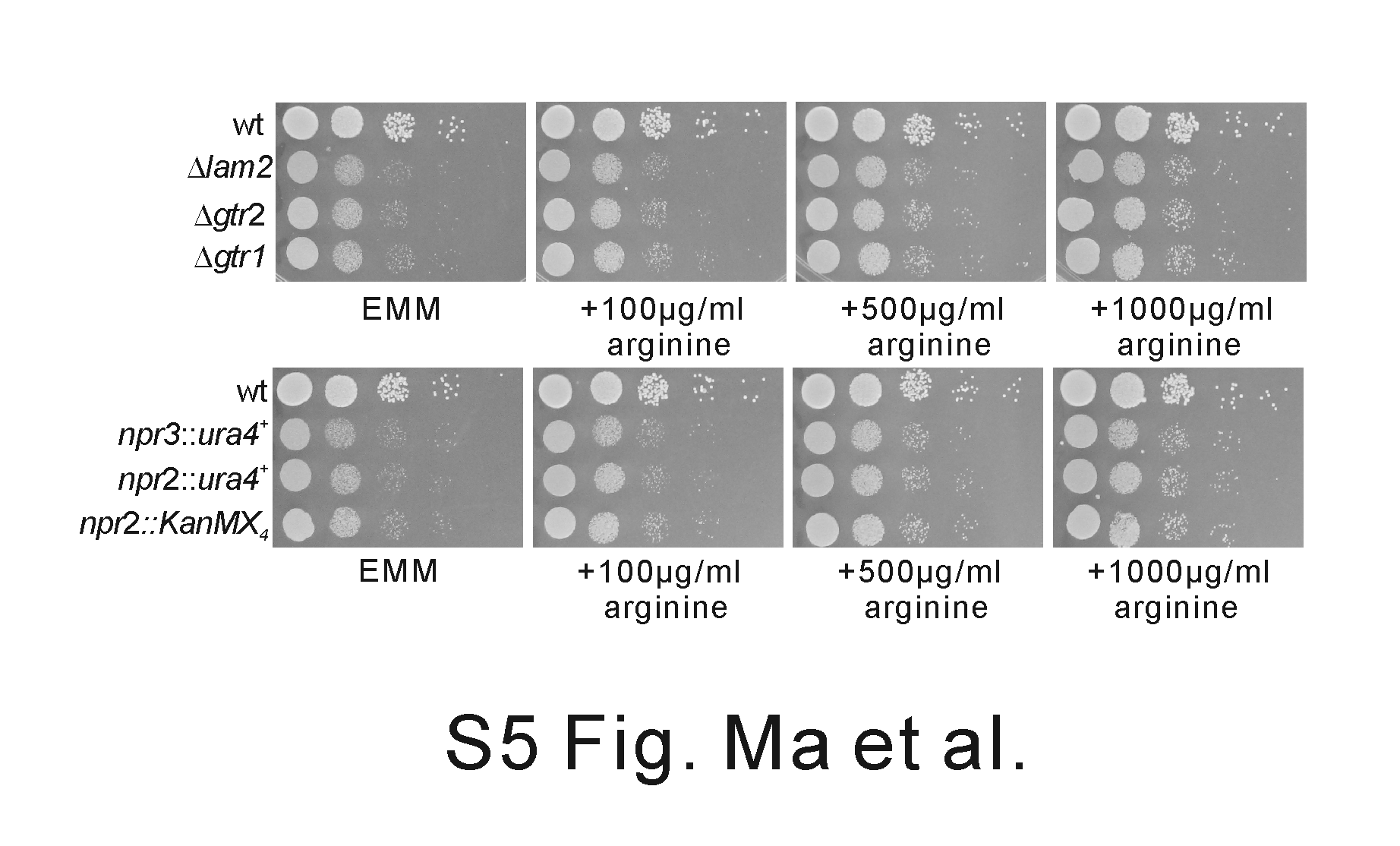

Supplement: S5 Fig — (TIF) [file pone.0156239.s005.tif]

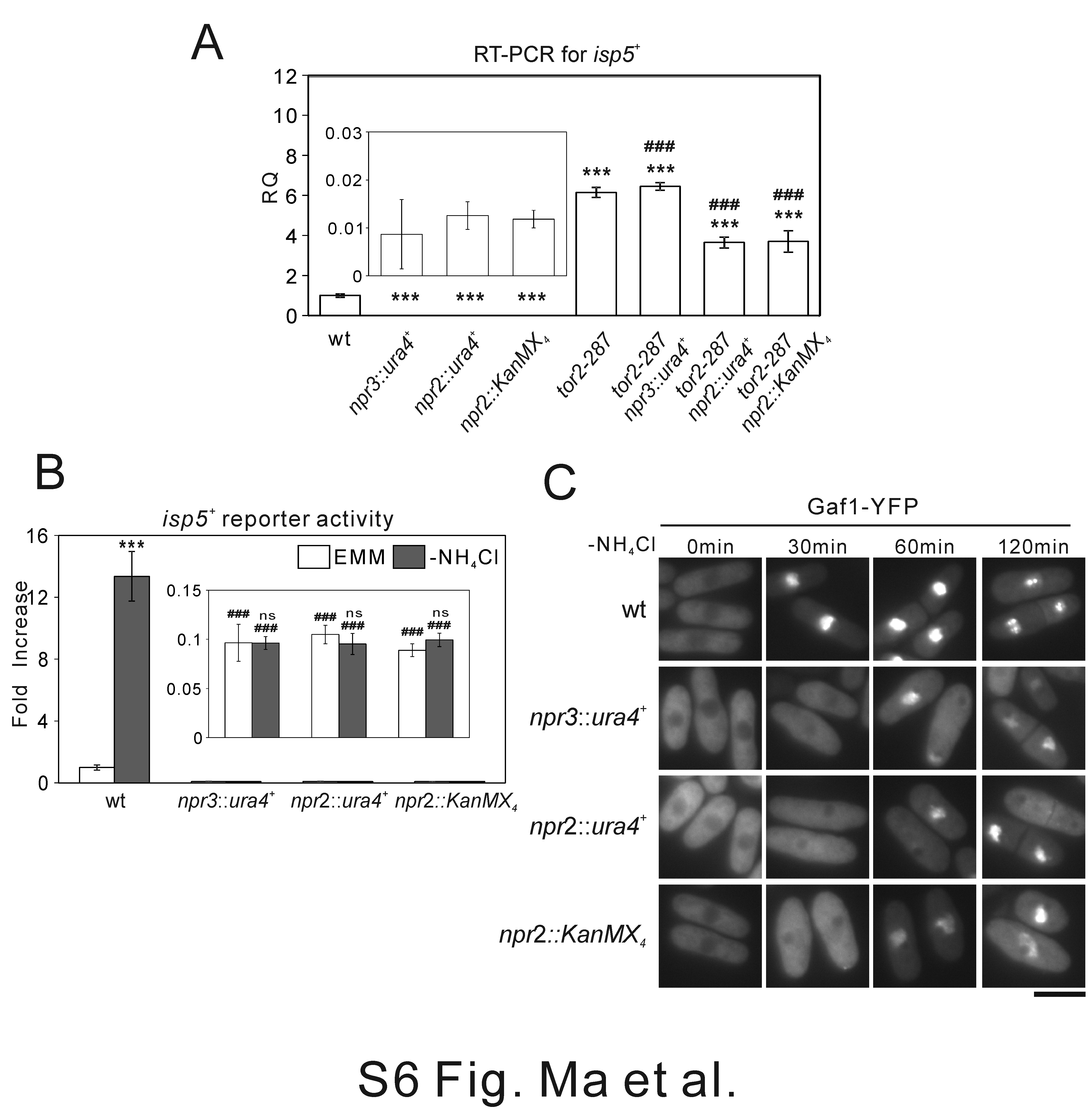

Supplement: S6 Fig — (TIF) [file pone.0156239.s006.tif]

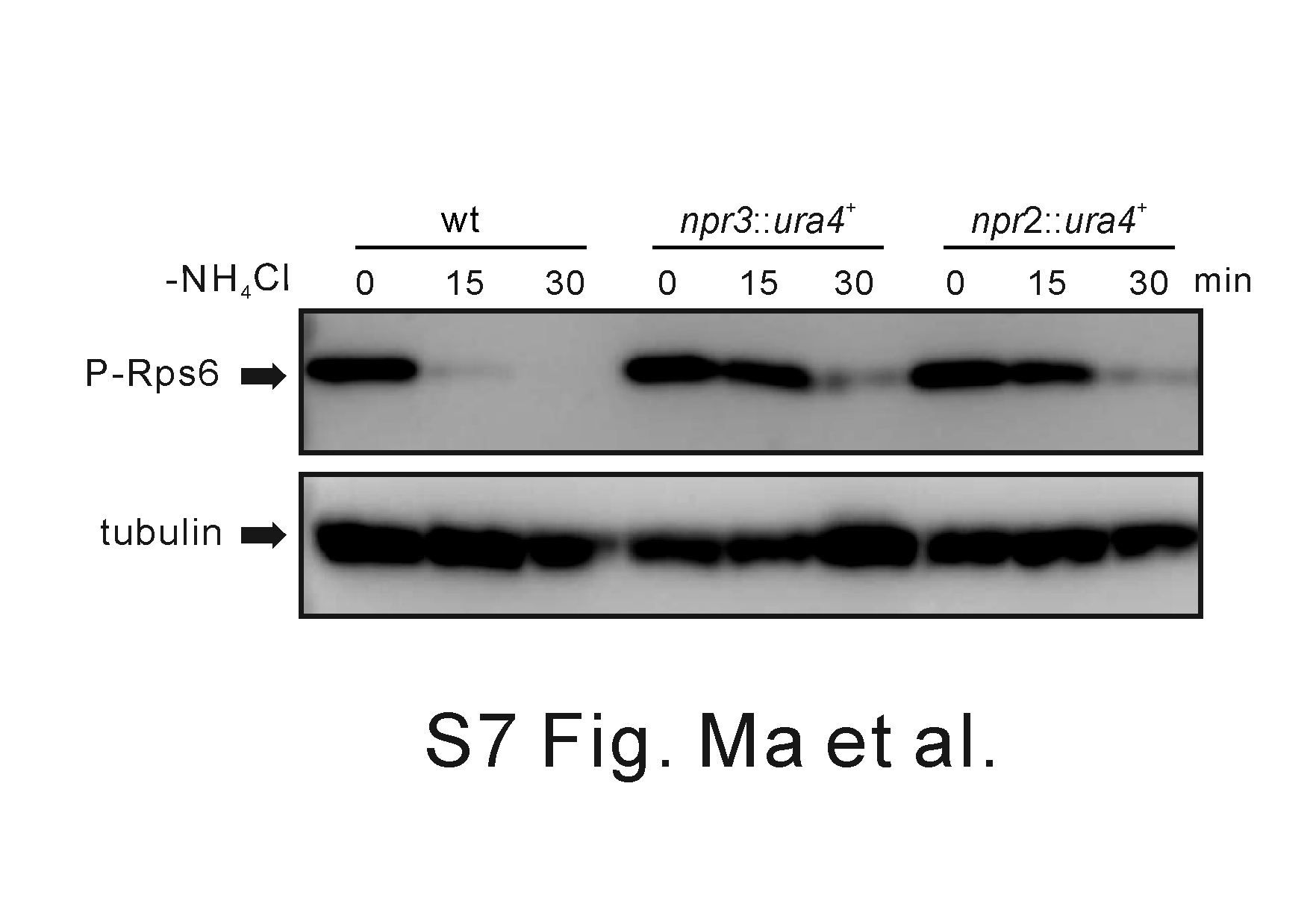

Supplement: S7 Fig — (TIF) [file pone.0156239.s007.tif]
