# Supplementary material for: The Loss of Lam2 and Npr2-Npr3 Diminishes the Vacuolar Localization of Gtr1-Gtr2 and Disinhibits TORC1 Activity in Fission Yeast
Source: PLoS One. 2016 May 26;11(5):e0156239. doi: 10.1371/journal.pone.0156239 (PMC4881991; doi:10.1371/journal.pone.0156239)
Supplement: S1 Table — (DOCX) [file pone.0156239.s008.docx]

**Supporting Information**

**Supplementary Materials and Methods**

**Gene Deletion**

*npr2*::*ura4*^+^ strain and *npr3*::*ura4*^+^ strain were generated using a PCR-based targeted gene deletion method as described previously [[1](#_ENREF_1)]. The fragment containing the *ura4*^+^ marker franked with the segments of the open reading frame from both ends (approximately 80 bp) was generated by PCR with the pKB3282 [[2](#_ENREF_2)] as a template. For *npr2*::*ura4*^+^, the sense primer was (4643) 5’-GGA GTA TTC TGA AGA GGG ATG GAT GGA TCA AGC TGA CAG CTT TCC GCC TCG TTT ATT GGC AAT ATT TTT TGC CCA ATA GGC CGA AAT CGG CAA AAT CCC-3’and the antisense primer was (4644) 5’-CAG GCA TCA CCC AAT CCA GCA ATC ATT TCA TTA ACA ACC TTT GGA GAC TTT TTC AAT GTG ACA CAC AAT TCA TCG GTG ATG GTT CAC GTA GTG GGC C-3’. For *npr3*::*ura4*^+^, the sense primer was (4712) 5’-ATG GTA CGC TTA ACT CCT CGA TTA GTA GCC ATT TTC CTT GTT GAA AAA ACA ACA TCA GGA GCT AAT TTC GTC CCA ATA GGC CGA AAT CGG CAA AAT CCC-3’ and the antisense primer was (4713) 5’-CCA AGA ATG CCA TCG AAG GAT GTA ATC ATT ATA CTC ATT AAG AAT ATT TCG CAT TAG TTT CCT TGG TAA GCC TTC GGT GAT GGT TCA CGT AGT GGG CC-3’. The amplified product was transformed into KP456 (*h^-^ leu1-32 ura4-D18*) and KP5079 (*h^-^ ura4-D18*). Stable integrants were selected on media lacking uracil, and disruption of the gene was checked by genomic PCR.

**Plasmids**

The plasmids to express the *npr2*^+^ and *npr3*^+^ genes under the respective promoters were generated using conventional methods. Briefly, the *npr2*^+^ or *npr3*^+^ gene was amplified by PCR with genomic DNA of wild-type cells as a template. For *npr2^+^*, the sense primer was (4509) 5’-CGG GAT CCA AAT ACT TTT ATT AAA ACT TC-3’ and the antisense primer was (3883) 5’-CGG GAT CCG CGG CCG CTC ATA CAT AAA TAA AAC AGG C-3’. For *npr3^+^*, the sense primer was (4969) 5’-CGG GAT CCT TCC TAT TTG CTA AAA ACT G-3’ and the antisense primer was (4977) 5’-CGG GAT CCG CGG CCG CTC ACC AAG AAT GCC ATC G-3’. The amplified product was digested with BamHI and NotI, and the resulting fragment was subcloned into the BamHI/NotI sites of a multicopy expression vector pKB1037. The resulted plasmids expressing the *npr2*^+^ and *npr3*^+^ genes are registered as pKB8726 and pKB9091, respectively.

**Legends to Supplementary Figures**

**S1 Fig. Graphical models for upstream regulators of TORC1 in mammals and yeasts. In mammals, the Ragulator complex composed of LAMTOR1, 2, 3, 4 and 5 functions as both a tether and a GEF for RagA or RagB. The GATOR1 complex composed of Nprl2, Nprl3 and DEPDC5 functions as a GAP for RagA or RagB. In budding yeast, the EGO complex composed of Ego1 and Ego3 together with Gtr1 and Gtr2 functions as positive regulators for TORC1, and Ego1 and Ego3 are thought to function similarly to LAMTOR1 and LAMTOR2-LAMTOR3, respectively. The SEACIT complex composed of Npr2, Npr3 and Iml1 functions as negative regulators via its GAP activity for Gtr1. In fission yeast, although Lam2, Npr2, Npr3 and Iml1 are homologs of LAMTOR2, Nprl2, Nprl3 and DEPDC5 in mammals, the functions of these proteins remain elusive. Dotted circles indicate unidentified homologs of components of the Ragulator and GATOR1 complexes.**

**S2 Fig. The deficits in Npr3 and Npr2 cause growth defect. (A)** Δ***npr3* and** Δ***npr2* cells showed growth defects. Prototrophic wild-type cells (wt, KP5080), *npr3*::*ura4^+^* cells (**KP6552**), *npr2*::*ura4^+^* cells (**KP6577**) and *npr2*::*KanMX*_4_ cells (**KP6506**) were spotted and incubated as described in Fig 1A**. **(B) Growth defects of** Δ***npr3* cells and** Δ***npr2* cells were rescued by overexpression of the respective genes. The leucine auxotrophic wild-type (wt, HM123),** *npr3*::*ura4*^+^ cells **(KP6551),** *npr2*::*ura4*^+^ **cells (KP6586) and** *npr2*::*KanMX_4_* **cells (KP6585) transformed with the control vector (pKB1037), or the plasmid containing the *npr3*^+^ (pKB9091) or *npr2*^+^ (pKB8726) gene were spotted and incubated as described in Fig. 1A.**

**S3 Fig.** Δ***npr3* cells and** Δ***npr2* cells show growth defects in a TORC1-dependent manner. (A) Growth defects of** Δ***npr3* cells and** Δ***npr2* cells were rescued by rapamycin or Torin1. The cells described as S2A Fig. were spotted onto the EMM, YES or YPD plates containing 0.2** μg/ml **rapamycin or 2** μM **Torin1 and incubated as described in Fig 1A. (B) Growth defects of** Δ***npr3* cells and** Δ***npr2* cells were rescued by *tor2-287* mutation.** Pr**ototrophic wild-type cells (KP5080, wt), *tor2-287* cells (**KP6214**), *npr3*::*ura4^+^* cells (**KP6552**), *tor2-287npr3*::*ura4^+^* cells (**KP6615**), *npr2*::*ura4^+^* cells (**KP6577**), *tor2-287npr2*::*ura4^+^* cells (**KP6674**), *npr2*::*KanMX*_4_ cells (**KP6506**) and *tor2-287npr2*::*KanMX_4_* cells (**KP6584**) were spotted and incubated as described in Fig 1A.**

**S4 Fig. The deficits in Npr3 and Npr2 increase the expression of *cat1*^+^ and Cat1 internalization in a TORC1-dependent manner.** (A) **mRNA levels of *cat1*^+^ were increased in** Δ*npr3* cells and Δ*npr****2* cells in a TORC1-dependent manner. The cells described as** in S3B Fig. were grown to mid-log phase in EMM medium. Total RNA was extracted from the harvested cells and subjected to quantitative RT-PCR for ***cat1*^+^** as described in Fig 3A. N = 3 for each group. ****P*<0.001 for Turkey’s test following one-way ANOVA for the comparisons with the value of wild-type cells. ^###^*P*<0.001 for Turkey’s test following one-way ANOVA compared with respective single knockout cells. (B) Cat1 internalization in **Δ*npr3* cells and Δ*npr2* cells was abolished upon pharmacological inhibition of TORC1. Wild-type cells (KP5859), *npr3*::*ura4^+^* cells (KP6614),** *npr****2*::*ura4^+^* cells (**KP6602**)** and ***npr2*::*KanMX*_4_ cells (**KP6603**) expressing** Cat1-GFP under its native promoter **were grown to mid-log phase in EMM medium. The cells were divided into two portions, one of which was treated with 0.2 μg/ml rapamycin and 10 mM caffeine for 60 min, and the other of which was left untreated. Representative fluorescent images of Cat1-GFP are shown. Scale bar, 10** μ**m. (C) Δ*npr3* cells and** Δ*npr****2* cells displayed canavanine resistance. The indicated cells as described in S2A Fig. were spotted onto EMM without or with canavanine at 60 or 90** μ**g/ml. The plates were incubated at 27**°C for 4 days without canavanine or for 5 days with canavanine. (D) **Δ*npr2* cells and** Δ*npr3* **cells showed canavanine resistance in a TORC1-dependent manner.** The indicated cells **as described in S2A Fig.** were **spotted onto 90** μ**g/ml canavanine without or with rapamycin or Torin. The plates were incubated at 27**°C for 4 days without canavanine or for 5 days with canavanine.

**S5 Fig. Exogenous addition of arginine did not rescue the defective cell growth of Δ*lam2* cells, Δ*gtr2* cells, Δ*gtr1* cells, Δ*npr3* cells and Δ*npr2* cells to the level of wild-type cells. Prototrophic wild-type cells (wt, KP5080), Δ*lam2* cells (**KP6578**), Δ*gtr1* cells (**KP6573**), Δ*gtr2* cells (**KP6571**), *npr3*::*ura4^+^* cells (**KP6552**), *npr2*::*ura4^+^* cells (**KP6577**) and *npr2*::*KanMX*_4_ cells (**KP6506**) were spotted onto EMM without or with arginine at 100** μ**g/ml, 500** μ**g/ml or 1000** μ**g/ml. The plates were incubated at 27**°C for 4 days.

**S6 Fig. Δ*npr3* cells and Δ*npr2* cells lack the basal transcription of *isp5*^+^ and its transcriptional activation associated with nuclear Gaf1 localization induced by nitrogen depletion. (A) mRNA levels of *isp5*^+^ were decreased in** Δ*npr3* cells and Δ*npr****2* cells in a TORC1-dependent manner. Total RNA from the indicated cells as described in S3B Fig. were** subjected to quantitative RT-PCR for *isp5*^+^ similarly to Fig 3A. N = 3 for each group. Magnified parts of the graphs are shown in insets. ********P*<0.001 for Turkey’s test following one-way ANOVA for the comparisons with the value of wild-type cells. ^###^*P*<0.001 for Turkey’s test following one-way ANOVA compared with respective single knockout cells. (B) Nitrogen depletion-induced *isp5*^+^ **transcriptional activation** was abolished in Δ*npr3* cells and Δ*npr****2*** cells. Wild-type cells (wt, HM123), ***npr3*::*ura4^+^* cells (**KP6551**), *npr2*::*ura4^+^* cells (**KP6586**) and *npr2*::*KanMX*_4_ cells (**KP6585**)** harboring the Renilla luciferase reporter plasmid for *isp5*^+^ promoter (pKB8527) were subjected to Renilla luciferase reporter assay, as described in Fig 4B. Magnified parts of the graphs are shown in insets. ********P*<0.001, ns not significant for unpaired *t*-test for the planned comparisons with the same genotype in the control condition. ^###^*P*<0.001 for Turkey’s test following one-way ANOVA compared with wild-type cells in the respective conditions (EMM or -NH_4_Cl). (C) Nuclear localization of Gaf1 upon nitrogen depletion was impaired in Δ*npr3* cells and Δ*npr2* cells. Wild-type cells (KP6488), ***npr3*::*ura4^+^* cells (**KP6613**), *npr2*::*ura4^+^* cells (**KP6599**) and *npr2*::*KanMX_4_* cells (**KP6604**)** expressing Gaf1-YFP under the native Gaf1 promoter were grown to mid-log phase in EMM medium. Fluorescent images of Gaf1-YFP were acquired before (0 min) or after nitrogen depletion (-NH_4_Cl) for the indicated time. Scale bar, 10 μm.

**S7 Fig.** **Nitrogen depletion-induced dephosphorylation of Rps6 is inhibited in Δ*npr3* cells and Δ*npr2* cells. Prototrophic wild-type cells (wt, KP5080), *npr3*::*ura4^+^* cells (**KP6552**) and *npr2*::*ura4^+^* cells (**KP6577**) were grown to mid-log phase in EMM medium. The cells were harvested before (0 min) or after nitrogen depletion (-NH_4_Cl) for 15 or 30 min. The cell lysates were subjected to immunoblot analysis for Rps6 phosphorylation (P-Rps6) as a readout for TORC1 activity.** α-Tubulin was detected as an internal control.

**S1 Table Fission yeast strains used in this study**

| **Strain** | **Genotype** | **Reference** |
| --- | --- | --- |
| KP5688 | *h^+^* | This study |
| KP5080 | *h^-^* | [[3](#_ENREF_3)] |
| KP456 | h^-^ *leu1-32 ura4-D18* | Our stock |
| HM123 | h^-^ *leu1-32* | Our stock |
| KP1248 | h^-^ *leu1-32 ura4-294* | Our stock |
| KP5079 | h^-^ *ura4-D18* | Our stock |
| KP6195 | h^+^ *ura4-D18* | Our stock |
| KP6214 | h^-^ *tor2-287* | [[3](#_ENREF_3)] |
| KP6552 | h^+^ *ura4 npr3*::*ura4*^+^ | This study |
| KP6577 | h^+^ *ura4 npr2*::*ura4*^+^ | This study |
| KP6506 | h^+^ *npr2*::*KanMX_4_* | This study |
| KP6594 | h^-^ *ura4 npr2*::*KanMX_4_* | This study |
| KP6615 | h^-^ *ura4 tor2-287 npr3*::*ura4^+^* | This study |
| KP6674 | h^+^ *ura4 tor2-287 npr2*::*ura4*^+^ | This study |
| KP6584 | h^+^ *tor2-287 npr2*::*KanMX_4_* | This study |
| KP6551 | h^+^ *leu1* *ura4 npr3*::*ura4*^+^ | This study |
| KP6586 | h^+^ *leu1 ura4 npr2*::*ura4*^+^ | This study |
| KP6585 | h^-^ *leu1 npr2*::*KanMX_4_* | This study |
| KP6595 | h^+^ *ura4 npr2*::*KanMX_4_* | This study |
| KP5859 | h^+^ *ura4 cat1-GFP*:*ura4*^+^ | [[3](#_ENREF_3)] |
| KP6614 | h^-^ *ura4 npr3*::*ura4^+^* *cat1-GFP*:*ura4*^+^ | This study |
| KP6602 | h^-^ *ura4 npr2*::*ura4^+^* *cat1-GFP*:*ura4*^+^ | This study |
| KP6603 | h^+^ *ura4 npr2*::*KanMX_4_ cat1-GFP*:*ura4*^+^ | This study |
| KP6488 | h^-^ *ura4* *gaf1-YFP*:*ura4*^+^ | [[4](#_ENREF_4)] |
| KP6613 | h^-^ *ura4 npr3*::*ura4^+^* *gaf1-YFP*:*ura4*^+^ | This study |
| KP6599 | h^+^ *ura4* *npr2*::*ura4*^+^ *gaf1-YFP*:*ura4*^+^ | This study |
| KP6604 | h^+^ *ura4* *npr2*::*KanMX_4_* *gaf1-YFP*:*ura4*^+^ | This study |
| KP92662 | h^+^ leu1-32 ura4-D18 ade6-M210/M216 *lam2*::*KanMX_4_* | [[5](#_ENREF_5)] |
| KP6578 | h^+^ *lam2*::*KanMX_4_* | This study |
| KP6571 | h^-^ *gtr2*::*hphMX* | This study |
| KP6573 | h^-^ *gtr1*::*KanMX* | This study |
| KP6636 | h^-^ *leu1* *ura4* *tet*O_7_*-*TATA*_CYC1_-*FLAG_3_*-gtr2*:*hphMX6*, | This study |
| KP6643 | h^-^ *leu1* *ura4* *tet*O_7_*-*TATA*_CYC1_-*FLAG_3_*-lam2*:*hphMX6* | This study |
| KP6641 | h^-^ *leu1 ura4* *tet*O_7_*-TATA_CYC1_-*FLAG_3_*-gtr2*:*hphMX6*, *pDM291-tet*R*-tup11*Δ*70*:*ura4*^+^ | This study |
| KP6648 | h^-^ *leu1* *ura4* *tet*O_7_*-TATA_CYC1_-*FLAG_3_*-lam2*:*hphMX6*, *pDM291-tet*R*-tup11*Δ*70*:*ura4*^+^ | This study |
| KP6645 | h^+^ *ura4* *tet*O_7_*-*TATA*_CYC1_-*FLAG_3_*-gtr2*:*hphMX6*, pDM291*-tet*R*-tup11*Δ*70*:*ura4*^+^ | This study |
| KP6650 | h^+^ *ura4* *tet*O_7_*-*TATA*_CYC1_-*FLAG_3_*-lam2*:*hphMX6*, pDM291*-tet*R*-tup11*Δ*70*:*ura4*^+^ | This study |
| KP6607 | h^-^ *leu1 lam2*::*KanMX_4_* | This study |
| KP6608 | h^-^ *leu1 gtr2*::*hphMX* | This study |
| KP6587 | h^+^ *tor2-287 lam2*::*KanMX_4_* | This study |
| KP6583 | h^+^ *tor2-287 gtr2*::*hphMX* | This study |
| KP6672 | h^+^ *tor2-287 gtr1*::*KanMX* | This study |
| KP6592 | h^-^ *ura4 lam2*::*KanMX_4_* | This study |
| KP6597 | h^+^ *ura4 gtr2*::*hphMX* | This study |
| KP6596 | h^-^ *ura4 gtr1*::*KanMX* | This study |
| KP5955 | h^+^ *ura4 tor2-287 cat1-GFP*:*ura4*^+^ | [[3](#_ENREF_3)] |
| KP6618 | h^+^ *ura4 tor2-287 lam2*::*KanMX_4_ cat1-GFP*:*ura4*^+^ | This study |
| KP6676 | h^+^ *ura4* *tor2-287 gtr2*::*hphMX cat1-GFP*:*ura4*^+^ | This study |
| KP6679 | h^+^ *ura4* *tor2-287 gtr1*::*KanMX cat1-GFP*:*ura4*^+^ | This study |
| KP6605 | h^+^ *ura4 lam2*::*KanMX_4_ cat1-GFP*:*ura4*^+^ | This study |
| KP6611 | h^+^ *ura4* *gtr2*::*hphMX cat1-GFP*:*ura4*^+^ | This study |
| KP6677 | h^+^ *ura4* *gtr1*::*KanMX cat1-GFP*:*ura4*^+^ | This study |
| KP6606 | h^+^ *ura4* *lam2*::*KanMX_4_* *gaf1-YFP*:*ura4*^+^ | This study |
| KP6610 | h^-^ *ura4* *gtr2*::*hphMX* *gaf1-YFP*:*ura4*^+^ | This study |
| KP6612 | h^-^ *ura4* *gtr1*::*KanMX* *gaf1-YFP*:*ura4*^+^ | This study |
| KP6588 | h^-^ *npr2*::*KanMX_4_* *gtr2*::*hphMX* | This study |
| KP6590 | h^-^ *lam2*::*KanMX_4_* *gtr2*::*hphMX* | This study |
| KP6682 | h^-^ *ura4* *npr2*::*ura4*^+^ *lam2*::*KanMX_4_* | This study |
| KP6683 | h^-^ *ura4* *npr3*::*ura4*^+^ *npr2*::*KanMX_4_* | This study |
| KP6684 | h^+^ *gtr2*::*hphMX gtr1*::*KanMX* | This study |
| KP6685 | h^-^ *ura4 gtr2*::*hphMX lam2*::*KanMX_4_* *gaf1-YFP*:*ura4*^+^ | This study |
| KP6686 | h^-^ *ura4 gtr2*::*hphMX npr2*::*KanMX_4_* *gaf1-YFP*:*ura4*^+^ | This study |
| KP6687 | h^-^ *ura4 gtr2*::*hphMX gtr1*::*KanMX* *gaf1-YFP*:*ura4*^+^ | This study |
| KP6192 | h*^-^ leu1-32 ura4-294 pREP1-GFP-Lam2*::*ura4^+^* | This study |
| KP6283 | h*^-^ leu1-32 ura4-294 pREP1-GFP-Npr2*::*ura4^+^* | This study |
| KP6688 | h^-^ *leu1-32 gtr1*::*KanMX* | This study |

**Supplementary References**

1. Bahler J, Wu JQ, Longtine MS, Shah NG, McKenzie A, 3rd, Steever AB, et al. Heterologous modules for efficient and versatile PCR-based gene targeting in *Schizosaccharomyces pombe*. Yeast. 1998;14(10):943-51. doi: 10.1002/(SICI)1097-0061(199807)14:10<943::AID-YEA292>3.0.CO;2-Y. PubMed PMID: 9717240.

2. Ma Y, Sugiura R, Saito M, Koike A, Sio SO, Fujita Y, et al. Six new amino acid-auxotrophic markers for targeted gene integration and disruption in fission yeast. Current genetics. 2007;52(2):97-105. doi: 10.1007/s00294-007-0142-1. PubMed PMID: 17622533.

3. Ma N, Liu Q, Zhang L, Henske EP, Ma Y. TORC1 signaling is governed by two negative regulators in fission yeast. Genetics. 2013;195(2):457-68. doi: 10.1534/genetics.113.154674. PubMed PMID: 23934889.

4. Ma Y, Ma N, Liu Q, Qi Y, Manabe R, Furuyashiki T. Tor Signaling Regulates Transcription of Amino Acid Permeases through a GATA Transcription Factor Gaf1 in Fission Yeast. PloS one. 2015;10(12):e0144677. doi: 10.1371/journal.pone.0144677. PubMed PMID: 26689777.

5. Kim DU, Hayles J, Kim D, Wood V, Park HO, Won M, et al. Analysis of a genome-wide set of gene deletions in the fission yeast *Schizosaccharomyces pombe*. Nature biotechnology. 2010;28(6):617-23. doi: 10.1038/nbt.1628. PubMed PMID: 20473289.
